# Community engagement in health guidelines and other normative products: a methodological review

**DOI:** 10.2471/BLT.24.292597

**Published:** 2025-09-29

**Authors:** Meghan A Bohren, Rana Islamiah Zahroh, Martha Vazquez Corona, Thiago M Santos, Andrew Booth, Mercedes Bonet, Ana Pilar Betrán, Özge Tunçalp

**Affiliations:** a Gender and Women’s Health Unit, Nossal Institute for Global Health, School of Population and Global Health, University of Melbourne, 207 Bouverie Street, Parkville, Victoria 3053, Australia.; b School of Medicine and Population Health, University of Sheffield, Sheffield, England.; c UNDP/UNFPA/UNICEF/WHO/World Bank Special Programme of Research, Development and Research Training in Human Reproduction, Geneva, Switzerland.; d Institute of Tropical Medicine Antwerp, Antwerp, Belgium.

## Abstract

**Objective:**

To explore and map how community engagement is conducted in the development or adaptation of health guidelines, norms and standards.

**Methods:**

We conducted a methodological review by searching MEDLINE, Scopus and CINAHL databases for articles published from January 2007 to May 2025, excluding publications reporting the engagement of only one or two community members on the guideline development panel. We extracted and categorized data on guideline characteristics (type of guideline, issuing entity, health-care topic), community engagement methods, the stages of guideline development for which community members were engaged and any evidence of evaluation. We compared the study characteristics using descriptive statistics.

**Findings:**

We reviewed 267 publications representing 258 unique studies, predominantly based in high-income countries. We observed that people affected by the health condition were most commonly engaged, and typically through surveys, workshops or as panel members. We noted that community engagement was most commonly used to identify community priorities and values, but less frequently for defining guideline scope or implementation. Although some studies described innovative approaches (for example, including lived-experience panels), these are rarely implemented globally. Only a small proportion of our reviewed studies included any evaluation of guideline development practices.

**Conclusion:**

Our review highlights the importance and challenges of implementing community engagement in global health guideline development, which may involve adapting existing engagement methods to a global context, leveraging technology while encouraging diversity, and carefully balancing the costs and benefits of extensive engagement. Striving for inclusive guideline development processes can lead to effective and equitable health recommendations worldwide.

## Résumé

**Objectif:**

Explorer et décrire la mise en œuvre de la participation communautaire dans l’élaboration ou l’adaptation de directives, normes et standards en matière de santé.

**Méthodes:**

Nous avons mené une revue méthodologique en effectuant des recherches dans les bases de données MEDLINE, Scopus et CINAHL pour trouver des articles publiés entre janvier 2007 et mai 2025, à l’exclusion des publications ne faisant état que de la participation d’un ou deux membres d’une communauté dans un panel d’élaboration des directives. Nous avons extrait et classé les données relatives aux caractéristiques des directives (type de directive, entité émettrice, thème de santé), aux méthodes de participation communautaire, aux étapes de l’élaboration des directives auxquelles les membres de la communauté ont participé et à toute preuve d’évaluation. Nous avons comparé les caractéristiques de l’étude à l’aide de statistiques descriptives.

**Résultats:**

Nous avons examiné 267 publications représentant 258 études distinctes, principalement menées dans des pays à revenu élevé. Nous avons observé que les personnes touchées par le problème de santé étudié présentaient souvent une participation élevée, généralement par le biais d’enquêtes, d’ateliers ou de panels. Nous avons noté que la participation communautaire permettait généralement d’identifier les priorités et les valeurs communautaires, mais moins fréquemment de définir la portée ou la mise en œuvre des directives. Bien que certaines études décrivent des approches innovantes (telles que l’inclusion de panels sur les expériences vécues), celles-ci sont rarement mises en œuvre à l’échelle mondiale. Seule une petite partie des études examinées comprenait une évaluation des pratiques d’élaboration des directives.

**Conclusion:**

Notre examen souligne l’importance et les défis de la mise en œuvre de la participation communautaire dans l’élaboration des directives mondiales en matière de santé. Cette situation peut impliquer d’adapter les méthodes de participation existantes au contexte mondial, de tirer parti de la technologie tout en encourageant la diversité et d’équilibrer soigneusement les coûts et les avantages d’une participation étendue. La recherche de processus d’élaboration de directives inclusives peut conduire à des recommandations sanitaires efficaces et équitables à l’échelle mondiale.

## Resumen

**Objetivo:**

Analizar y describir cómo se lleva a cabo la participación comunitaria en el desarrollo o la adaptación de guías, normas y estándares en el ámbito de la salud.

**Métodos:**

Se realizó una revisión metodológica mediante búsquedas en las bases de datos MEDLINE, Scopus y CINAHL de artículos publicados entre enero de 2007 y mayo de 2025, excluyéndose las publicaciones que informaban de la participación de solo uno o dos miembros de la comunidad en el panel de elaboración de la guía. Se extrajeron y categorizaron datos sobre las características de las guías (tipo, entidad emisora, tema de atención sanitaria), los métodos de participación comunitaria, las etapas del desarrollo de las guías en las que participaron miembros de la comunidad y cualquier evidencia de evaluación. Las características de los estudios se compararon utilizando estadística descriptiva.

**Resultados:**

Se revisaron 267 publicaciones correspondientes a 258 estudios únicos, la mayoría de ellos realizados en países de ingresos altos. Se observó que las personas afectadas por la enfermedad o condición de salud eran las más frecuentemente involucradas, generalmente a través de encuestas, talleres o como miembros de los paneles. La participación comunitaria se utilizó principalmente para identificar las prioridades y valores de la comunidad, aunque con menor frecuencia para definir el alcance de las guías o su implementación. Aunque algunos estudios describieron enfoques innovadores (por ejemplo, paneles de personas con experiencia vivida), estos se aplican raramente a nivel mundial. Solo una pequeña proporción de los estudios revisados incluía alguna forma de evaluación de las prácticas de elaboración de guías.

**Conclusión:**

Esta revisión pone de relieve la importancia y los desafíos de aplicar la participación comunitaria en la elaboración de guías de salud a nivel mundial, lo que puede implicar adaptar los métodos existentes a contextos globales, aprovechar la tecnología fomentando la diversidad y equilibrar cuidadosamente los costos y beneficios de una participación amplia. Promover procesos de elaboración de guías inclusivos puede conducir a recomendaciones sanitarias más eficaces y equitativas en todo el mundo.

## ملخص

الغرض استكشاف ورسم خريطة لكيفية إشراك المجتمع في تطوير أو تكييف المبادئ التوجيهية والمعايير والمقاييس الصحية.

الطريقة قمنا بإجراء مراجعة منهجية من خلال البحث في قواعد بيانات MEDLINE، وScopus، وCINAHL عن المقالات المنشورة من يناير/كانون ثاني 2007 إلى مايو/أيار 2025، باستثناء المنشورات التي أفادت بمشاركة عضو واحد أو اثنين فقط من أعضاء المجتمع في لجنة تطوير المبادئ التوجيهية. قمنا باستخراج وتصنيف البيانات بناءً على خصائص المبادئ التوجيهية (نوع المبادئ التوجيهية، الجهة المُصدرة، موضوع الرعاية الصحية)، وأساليب إشراك المجتمع، ومراحل تطوير المبادئ التوجيهية التي شارك فيها أعضاء المجتمع، وأي دليل على التقييم. قمنا بمقارنة خصائص الدراسة باستخدام الإحصاءات الوصفية.

النتائج قمنا بمراجعة 267 منشورًا تُمثل 258 دراسة فريدة، معظمها في دول ذات دخل مرتفع. لاحظنا أن الأشخاص المتأثرين بالحالة الصحية هم الأكثر مشاركة، وعادةً ما يكون ذلك من خلال الاستبيانات، أو ورش العمل، أو كأعضاء في اللجنة. وقد لاحظنا أن المشاركة المجتمعية تُستخدم غالبًا لتحديد أولويات المجتمع وقيمه، ولكنها تُستخدم بشكل أقل لتحديد نطاق المبادئ التوجيهية أو تنفيذها. على الرغم من أن بعض الدراسات وصفت مناهجًا مبتكرة (على سبيل المثال، بما يشمل لجان التجارب المعاشة)، إلا أنها نادرًا ما تُنفذ عالميًا. فقط نسبة ضئيلة من دراساتنا التي راجعناها تضمنت تقييمًا لممارسات تطوير المبادئ التوجيهية.

الاستنتاج تُسلط مراجعتنا الضوء على أهمية المشاركة المجتمعية وتحديات تنفيذها في تطوير المبادئ التوجيهية الصحية العالمية، والتي قد تشمل تكييف أساليب المشاركة الحالية مع السياق العالمي، والاستفادة من التكنولوجيا مع تشجيع التنوع، والموازنة الدقيقة بين تكاليف المشاركة المكثفة وفوائدها. إن السعي إلى عمليات شاملة لتطوير المبادئ التوجيهية يمكنه أن يؤدي إلى توصيات صحية فعالة ومنصفة في جميع أنحاء العالم.

## 摘要

**目的:**

旨在探讨和了解如何在卫生指南、规范和标准制定或修订过程中实现社区参与。

方法 我们利用美国联机医学文献分析和检索系统 (MEDLINE)、Scopus 以及护理和联合卫生文献累积索引 (CINAHL) 数据库搜索了 2007 年 1 月至 2025 年 5 月期间出版的相关文章（未将表明仅一个或两个社区成员加入指南制定小组的出版物计入搜索结果），并开展了方法论评价。我们提取了涉及指南特征（指南类型、发行单位、卫生保健主题）、社区参与方法、社区成员参与的指南制定阶段以及任何评估依据的相关数据，并对此类数据进行了归类。我们采用描述性统计方法对研究特征进行了对比。

**结果:**

我们审查了 267 篇介绍主要基于高收入国家开展的 258 项特殊研究的出版物。我们发现，最常见的参与者是受健康问题困扰的人群，他们通常通过调查、研讨会或以小组成员的身份参与其中。我们注意到，社区参与最常用于确定社区的优先事项和价值观，但较少用于界定指南的适用范围或实施方式。尽管部分研究对创新方法（例如：将亲身体验专家小组纳入其中）进行了描述，但在全球范围内却很少推行这些方法。在我们审查的研究中，仅小部分研究对指南制定实践进行了评估。

**结论:**

我们的审查结果凸显了在全球卫生指南制定过程中实现社区参与的重要性以及所面临的挑战，为了应对此类挑战，可能需要调整现有参与方法以使其适用于全球各个国家、在充分利用各项技术的同时鼓励多元化发展，并谨慎权衡广大参与群体产生的成本和效益。致力于推出具有包容性的指南制定流程，从而在全球范围内提供切实有效且公平合理的卫生建议。

## Резюме

**Цель:**

Изучить и зафиксировать то, каким образом осуществляется вовлечение общественности в разработку или адаптацию рекомендаций по здравоохранению, норм и стандартов.

**Методы:**

Авторы выполнили методологический обзор, проведя по базам данных MEDLINE, Scopus и CINAHL поиск статей, опубликованных в период с января 2007 года по май 2025 года. В обзор не вошли публикации, в которых сообщалось о привлечении одного-двух представителей общественности в группу разработки рекомендаций. Были собраны и категоризированы данные по характеристикам рекомендаций (по типу, издающему органу, тематике в здравоохранении), а также по методам вовлечения общественности, стадиям разработки рекомендаций, на которых привлекались представители общественности, и по любым доступным доказательствам проведения оценки. Для сравнения характеристик исследования использовались методы дескриптивной статистики.

**Результаты:**

В обзор было включено 267 публикаций, представляющих 258 уникальных исследований, в основном из стран с высоким уровнем дохода населения. По наблюдениям авторов, чаще всего к работе привлекались люди, страдающие теми или иными заболеваниями, в основном через анкетирование, проведение семинаров или участие в качестве членов рабочих групп. Было отмечено, что представителей общественности привлекали к работе чаще всего для определения общественных приоритетов и ценностей и реже для определения объема рекомендаций или их внедрения. Хотя в некоторых исследованиях был продемонстрирован инновационный подход (например, включение рабочих групп с опытом жизни с тем или иным заболеванием), в мировом масштабе это все еще редкое явление. Лишь в незначительной части исследований, включенных в обзор, проводилось хотя бы минимальное оценивание практик разработки рекомендаций.

**Вывод:**

Данный обзор подчеркивает важность активного вовлечения общественности в разработку глобальных рекомендаций в области здравоохранения и соответствующие проблемы, для решения которых может понадобиться адаптация существующих методов вовлечения в глобальном контексте, использование технологий и в то же время поощрение разнообразия при одновременном тщательном соблюдении баланса затрат на такое вовлечение и получаемой пользы. Стремление к реализации инклюзивного процесса разработки может привести к появлению эффективных и справедливых рекомендаций по здравоохранению во всем мире.

## Introduction

A variety of scientific and technical normative publications exist in the field of health, with different target audiences, processes and development methods. These include recommendations on how to provide care, and are presented as standards, guidelines, implementation guidance, knowledge gaps, classifications and nomenclatures, strategies and policies, learning materials and evaluation methods. Developed to support Member State ministries, health systems, health workers and the public to achieve optimal health outcomes, normative publications should follow an evidence-based, high-quality and transparent process.

Of all normative documents, guidelines are among the best established in terms of the development process. Well-conducted, evidence-based guidelines typically summarize the evidence of the effectiveness of interventions or systems, alongside values, acceptability, feasibility, equity and human rights, and implementation considerations. Engaging stakeholders is critical to ensure that the perspectives of guideline end-users are addressed appropriately, and that guidelines are relevant, transparent and useful to those affected by them.^1^ Ten stakeholder groups have been described in health and health-care guideline development: patients and caregivers, payers, purchasers, journal editors, policy-makers, research principal investigators, product makers, programme managers, health workers and the public.^2^ Historically, health workers, policy-makers and programme managers have contributed most substantially to guideline development. More recently, however, people with lived experience of the health condition, patients, carers and/or advocacy groups have been engaged in the development process, which has the potential to democratize health policy development and decision-making.^3^ Engaging with groups historically marginalized from health-care decision-making, such as those experiencing social disadvantage or exclusion from primary research included in a guideline, can improve equity^3^ by ensuring that their perspectives are reflected in recommendations and policies affecting their health and well-being.

Many related terms are used to describe the development of guidelines with and/or by members of the public, including patient engagement, patient and public involvement, patient and public involvement and engagement, and consumer engagement. We use the term community engagement in this publication to provide a holistic view of health (e.g. those engaged do not need to be a patient or consumer of health-care services), and define community engagement as involving people with the health condition or using the health-care service, their caregivers or the public.^2^

Setting global norms and standards, including developing guidelines, is a core function of the World Health Organization (WHO). Direct engagement with relevant stakeholders (specifically, community engagement in WHO normative activities) has the potential to: enhance ownership of recommendations; strengthen effective translation into policy, practice and impact; build public confidence in guidelines; and improve the relevance, usefulness and person-centredness of guidelines. In 2021, the Human Reproduction Programme/WHO Gender and Rights Advisory Panel^4^ recommended more meaningful community engagement in guideline prioritization, development, dissemination and implementation.

Substantial progress in improving community engagement in research priority setting, primary research and systematic reviews has been made in the last decade,^5^ with much to learn from these processes. However, less is known about best practices for community engagement in the development of guidelines, norms and standards, and how to ensure meaningful community engagement at a global level. To address these knowledge gaps we conducted a methodological review (which is designed to map and summarize methodological practices of research,^6^^,^^7^ and to identify areas for innovation and improvement) to explore how communities can be engaged in the development and/or adaptation of health guidelines. 

## Methods

In the absence of reporting guidance for methodological reviews, we conducted our review according to the Preferred Reporting Items for Systematic Reviews and Meta-Analyses extension for scoping reviews (PRISMA-ScR).^8^ We registered our protocol on the Open Science Framework.^9^

### Inclusion and exclusion criteria

Our topics of interest included current practices of community engagement in the development of health guidelines, norms and standards, regardless of health topic or geographical location. We included evidence about community engagement at any stage of guideline development, according to the definition of stages in the *WHO handbook for guideline development*:^10^ scope guideline; create guideline panel and external review group; formulate population, intervention, comparison and outcome questions and outcomes; evidence retrieval, assessment and synthesis; appraise certainty in the body of evidence; formulate recommendations; disseminate and implement; and evaluate impact. We included methods literature (e.g. case studies of methods used for community engagement), reports of guideline development or adaptation processes, and guideline documents without any restrictions in terms of study design, language, country or setting. To ensure the consideration of relevant data on contemporary guideline development, we only considered publications from 2007 onwards that were available as full text.

We excluded publications that reported engagement only as one or two members of the guideline development group from further analyses because: (i) they provided no additional information on innovative methods for community engagement; (ii) the advantages and limitations of this type of involvement are already well established; (iii) such publications were deprioritized by a panel of women’s health advocates; and (iv) the high volume of publications. 

### Search methods and study selection

We searched the databases MEDLINE, Scopus and CINAHL from 1 January 2007 to 9 May 2025, combining concepts of “guideline development” and “community engagement” (see online repository for search terms).^11^ We imported database search results into Covidence (Veritas Health Innovation, Melbourne, Australia). At least two reviewers independently reviewed title and abstracts. We used Google Translate to translate titles and abstracts published in languages other than those in which the review team are proficient. We retrieved full texts of all potentially relevant publications and two reviewers assessed eligibility independently. We resolved any disagreements through discussion with a third reviewer.

### Data extraction and synthesis

Our reviewed publications either described substantive community engagement (e.g. a lived-experience advisory panel) or were methods papers describing how to engage the community in guideline development. Two reviewers used a purpose-designed and tested data extraction form to extract information on: setting; design; sample size and characteristics; data collection and analysis; type of guidelines (topic, method); guideline-issuing entity; community engagement and methods used; scope of engagement; and recommendations for best practices or lessons learnt. Disagreements were resolved through discussion with a third reviewer. We followed methodological review methods by generating an overview of strategies for community engagement in guideline development, without requiring quality assessment.

During the charting stage of the synthesis, we coded extracted data into meaningful categories for comparison across publications. We coded publications as: guideline development; guideline adaptation and/or translation: primary research for guideline development; primary research about community engagement in guidelines; guideline development protocols; guideline methods; reviews of guidelines or commentaries on guidelines. We coded the target population as: people affected by the guideline, caregivers, public or guideline developers. We coded health system level to reflect the target setting or audience for the guideline, namely: facility, national, regional, multicountry or global. We coded health topics based on the International Statistical Classification of Diseases and related health problems, 11th revision (ICD-11). We classified publications related to patient care or health promotion without a specific health condition (for example, intensive care unit or vaccination) as general patient care and health promotion, and publications across multiple ICD chapters or without specific health topics as multiple conditions or not specified. We combined categories of less than five publications as other. We coded stages of community engagement in guideline development as: define guideline scope; describe community priorities, values, experiences or preferences; review evidence and/or formulate recommendations; review drafted recommendations; create community version of guidelines; and implement and/or disseminate guidelines.

We described characteristics of included publications using absolute and relative frequencies and presented these in plots using R version 4.1.1 (RFfS Computing, Vienna, Austria). We presented and interpreted preliminary review results at a workshop convened by WHO in May 2024 and hosted by the Brocher Foundation, titled *Women and civil society engagement for meaningful and relevant global research, norms and standards on maternal and perinatal health*, and attended by 32 participants from civil societies and universities. We considered workshop discussions when completing this review, focusing on: (i) an analysis of studies with creative approaches to substantive community engagement, rather than describing those studies that simply included one or two community members on the guideline development group; and (ii) community engagement approaches that create and offer safe environments for sharing and challenging power.

## Results

**Fig. 1:**
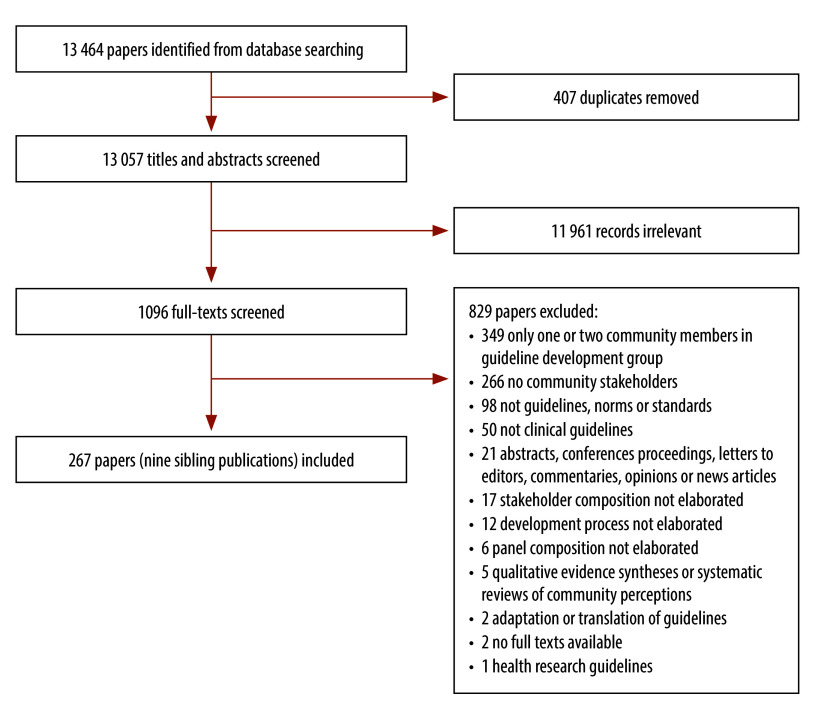
Flowchart depicting the selection of articles on community engagement in guideline development

We identified 13 464 citations from the database searches, and included 267 publications in our review^2^^,^^12^^–^^277^ (Fig. 1; Table 1 available at: https://www.who.int/publications/journals/bulletin). A list of excluded publications is available in the online repository.^11^ Because our list of 267 reviewed publications includes nine pairs of publications reporting on the same study, we present our results in terms of 258 unique studies.

**Table 1:** Characteristics of the included studies on community engagement in health guidelines normative products

Author, year; reference	Country and/or region	Type of study	Guideline issuing entity (type of issuing entity)	Guideline topic (based on ICD-11)	Guideline health system level	No. of community members involved
Langlands et al., 2008^143^	Australia, Canada, Ireland, New Zealand, United Kingdom, USA	Guideline development	Orygen Health Research Centre (nonprofit)	Mental, behavioural or neurodevelopmental disorders	National	> 100
Langlands et al., 2008^144^	Australia, Canada, New Zealand, United Kingdom, USA	Guideline development	Orygen Health Research Centre (nonprofit)	Mental, behavioural or neurodevelopmental disorders	National	> 100
Lee et al., 2008^148^	Republic of Korea	Qualitative research (part of guideline)	NR	Patient care	National	21–30
Ajayi et al., 2009^14^	Nigeria	Guideline development	NR	Certain infectious or parasitic diseases	Regional	31–40
Levin et al., 2009^152^	USA	Guideline development	Multiple organizations (collaboration of various types of organizations)	Certain infectious or parasitic diseases	Regional	NR and/or NA
Boivin et al., 2010^39^	Australia, Belgium, Canada, Czechia, Finland, France, Germany, Japan, Netherlands (Kingdom of the), New Zealand, Norway, Spain, United Kingdom, USA	Guideline development	Guideline International Network Patient and Public Involvement Working Group (nonprofit)	Other	Multicountry	NR and/or NA
Kelly et al., 2010^129^	Australia, Canada, New Zealand, United Kingdom, USA	Guideline development	Orygen Health Research Centre (nonprofit)	Mental, behavioural or neurodevelopmental disorders	Multicountry	11–20
Sims et al., 2010^219^	Australia	Guideline development	Australian Government Department of Health and Ageing (government body)	Prevention	National	NR and/or NA
Westby & Backman, 2010^264^	Canada, USA	Qualitative research (part of guideline)	NR	Diseases of the musculoskeletal system or connective tissue	Multicountry	31–40
Berk et al., 2011^32^	Australia	Guideline development	Orygen Health Research Centre (nonprofit)	Mental, behavioural or neurodevelopmental disorders	National	51–100
Díaz del Campo et al., 2011^70^	Spain	Guideline development	NR	Multiple conditions	National	11–20
Franx et al., 2011^88^	Netherlands (Kingdom of the)	Guideline development	National Steering Group for Multidisciplinary Guideline Development in Mental Health (research or guideline consortium)	Mental, behavioural or neurodevelopmental disorders	National	2–10
Harding et al., 2011^108^	United Kingdom	Review	National Collaborating Centre for Mental Health (nonprofit)	Mental, behavioural or neurodevelopmental disorders	National	NR and/or NA
Kingston et al., 2011^134^	Australia, Canada, New Zealand, United Kingdom, USA	Guideline development	Orygen Health Research Centre (nonprofit)	Mental, behavioural or neurodevelopmental disorders	Multicountry	51–100
Légaré et al., 2011^149^	Australia, Germany, United Kingdom, USA	Review	Guideline International Network Patient and Public Involvement Working Group (nonprofit)	Multiple conditions	Multicountry	NR and/or NA
O'Brien et al., 2011^179^	Canada, United Kingdom	Qualitative research (part of guideline)	NR	Certain infectious or parasitic diseases	Multicountry	NR and/or NA
Tremblay et al., 2011^247^	Canada	Guideline development	Canadian Society for Exercise Physiology (professional)	Prevention	National	NR and/or NA
Cluzeau et al., 2012^52^	USA and multiple European countries (NR)	Method development	Multiple organizations (professional)	Diseases of the respiratory system	Multicountry	NR and/or NA
den Breejen et al., 2012^66^	Netherlands (Kingdom of the)	Guideline development	Multiple organizations (collaboration of various types of organizations)	Diseases of the genitourinary system	National	> 100
Dunning et al., 2012^76^	Australia	Guideline development	NR	Endocrine, nutritional or metabolic diseases	NR	21–30
Flynn et al., 2012^83^	Ireland	Quantitative research (part of guideline)	Food Safety Authority of Ireland (government body)	Prevention	National	> 100
Foulon et al., 2012^84^	Canada	Guideline development	Spinal Cord Injury Action Canada (nonprofit)	Injury, poisoning or certain other consequences of external causes	National	51–100
Hudson et al., 2012^118^	Australia	Guideline development	Centre for Palliative Care, St Vincent Hospital (hospital)	Mental, behavioural or neurodevelopmental disorders	National	2–10
Kelson et al., 2012^130^	USA and multiple European countries (NR)	Method development	Multiple organizations (professional)	Diseases of the respiratory system	Multicountry	NR and/or NA
Kunz et al., 2012^140^	USA and multiple European countries (NR)	Method development	Multiple organizations (professional)	Diseases of the respiratory system	Multicountry	NR and/or NA
Schrijvers et al., 2012^216^	Multiple European countries (NR)	Quantitative research (patient engagement in guidelines)	European Cancer Organisation (nonprofit)	Neoplasms	Multicountry	NR and/or NA
Tong et al., 2012^240^	Australia	Guideline development	Caring for Australians with Renal Impairment (research or guideline consortium)	Diseases of the genitourinary system	National	21–30
Tremblay et al., 2012^245^	Canada	Guideline development	Canadian Society for Exercise Physiology (professional)	Prevention	National	NR and/or NA
Tremblay et al., 2012^246^	Canada	Guideline development	Canadian Society for Exercise Physiology (professional)	Prevention	National	NR and/or NA
WHO, 2012^270^	Brazil, China, Côte d’Ivoire, Iran (Islamic Republic of), Sri Lanka, Ukraine	Guideline development	World Health Organization (international organization)	Certain infectious or parasitic diseases	Global	51–100
den Breejen et al., 2013^67^	Netherlands (Kingdom of the)	Guideline development	NR	Diseases of the genitourinary system	National	11–20
Dunning et al., 2013^75^; see also Dunning et al., 2012^76^	Australia	Guideline development	NR	Endocrine, nutritional or metabolic diseases	NR	21–30
Porcheret et al., 2013^199^	United Kingdom	Guideline development	NR	Diseases of the musculoskeletal system or connective tissue	National	11–20
Taruscio et al., 2013^234^	Bulgaria, Czechia, France, Greece, Netherlands (Kingdom of the), Portugal, Slovakia, Slovenia	Guideline development	European Project for Rare Diseases National Plans Development (research or guideline consortium)	Multiple conditions	Multicountry	NR and/or NA
Tuffrey-Wijne et al., 2013^250^	United Kingdom	Qualitative research (part of guideline)	NR	Multiple conditions	National	41–50
Boelens et al., 2014^38^	Italy, Netherlands (Kingdom of the), Spain, United Kingdom	Guideline development	Multiple organizations (professional)	Neoplasms	Multicountry	NR and/or NA
den Breejen et al., 2014^68^; see also den Breejen et al., 2012^66^	Netherlands (Kingdom of the)	Guideline development	Multiple organizations (collaboration of various types of organizations)	Diseases of the genitourinary system	National	> 100
García-Toyos et al., 2014^89^	Spain	Qualitative research (part of guideline)	NR	Patient care	Regional	41–50
Roman & Feingold, 2014^206^	USA	Commentary	Multiple organizations (professional)	Multiple conditions	National	NR and/or NA
Wolfe et al., 2014^268^	USA	Guideline development	American Family Children’s Hospital (hospital)	Patient care	Facility	NR and/or NA
Beanland et al., 2015^28^	Global (Ghana, other countries NR)	Guideline development	World Health Organization	Certain infectious or parasitic diseases	Global	31–40
Calvo et al., 2015^47^	Spain	Guideline development	Spanish Society of Pediatric Rheumatology (professional)	Diseases of the musculoskeletal system or connective tissue	National	NR and/or NA
DeMatteo et al., 2015^63^	Canada	Guideline development	MacMaster University (academic)	Injury, poisoning or certain other consequences of external causes	National	NR and/or NA
Holloway et al., 2015^116^	Australia	Qualitative research (part of guideline)	Australian Government Department of Health and Ageing (government body)	Patient care	National	11–20
Monaghan-Bem et al., 2015^170^	USA	Guideline development	Children’s Hospital, Pediatrics NYC, Mount Sinai (hospital)	Patient care	Facility	11–20
O'Reilly-de Brún et al., 2015^180^	Ireland	Qualitative research (part of guideline)	Health Service Executive (government body)	Patient care	National	51–100
Pittens et al., 2015^196^	Netherlands (Kingdom of the)	Guideline development	VU University Medical Center, Amsterdam (hospital)	Diseases of the genitourinary system	National	21–30
Serrano-Aguilar et al., 2015^218^	Spain	Guideline development	NR	Diseases of the immune system	National	> 100
Taddio & Rogers, 2015^233^	Canada	Guideline development	Help Eliminate Pain in Kids and Adults (HELPinKIDS; nonprofit)	Patient care	National	NR and/or NA
van de Bovenkamp et al., 2015^252^	Netherlands (Kingdom of the)	Review	Dutch Council for Quality of Healthcare (government body)	Other	National	NR and/or NA
van der Ham et al., 2015^253^	Netherlands (Kingdom of the)	Guideline development	Not specified	Mental, behavioural or neurodevelopmental disorders	National	21–50
Wiener et al., 2015^266^	USA	Guideline development	Mattie Miracle (patient advocacy group)	Mental, behavioural or neurodevelopmental disorders	National	2–10
Zuidema et al., 2015^277^	Netherlands (Kingdom of the), Norway, United Kingdom	Guideline development	NR	Mental, behavioural or neurodevelopmental disorders	Multicountry	21–30
Bond et al., 2016^40^	Australia	Guideline development	Mental Health First Aid Australia (nonprofit)	Mental, behavioural or neurodevelopmental disorders	National	31–40
Fraenkel et al., 2016^86^	USA	Guideline development	American College of Rheumatology (professional)	Diseases of the musculoskeletal system or connective tissue	National	11–20
Hämeen-Anttila et al., 2016^106^	Finland	Qualitative research (patient engagement in guidelines)	Finnish Medical Society Duodecim (professional)	NR and/or NA	National	11–20
Healy & Gillen, 2016^112^	Ireland	Guideline development	Guidelines and Audit Implementation Network (research or guideline consortium)	Patient care	National	NR and/or NA
Kobleder et al., 2016^136^	NR	Guideline development	NR	Neoplasms	Multicountry	NR and/or NA
Lindsay et al., 2016^155^	Canada	Qualitative research (part of guideline)	NR	Injury, poisoning or certain other consequences of external causes	National	11–20
Pai et al., 2016^186^	Canada	Guideline development	National Hemophilia Foundation (nonprofit)	Diseases of the blood or blood-forming organs	National	2–10
Schleedoorn et al., 2016^214^	Belgium, Finland, Germany, Ireland, Israel, Netherlands (Kingdom of the), Portugal, Sweden, United Kingdom	Guideline development	European Society of Human Reproduction and Embryology (professional)	Diseases of the genitourinary system	Multicountry	2–10
Tong et al., 2016^241^	Australia	Qualitative research (part of guideline)	Kidney Health Australia (nonprofit)	Diseases of the genitourinary system	National	11–20
Tremblay et al., 2016^243^	Canada	Guideline development	Canadian Society for Exercise Physiology (nonprofit)	Prevention	National	NR and/or NA
Armstrong & Bloom, 2017^19^	USA	Review	Multiple organizations (collaboration of various types of organizations)	NR and/or NA	NR	NR and/or NA
Armstrong et al., 2017^21^	USA	Qualitative research (patient engagement in guidelines)	NA	NR and/or NA	NR	11–20
Armstrong et al., 2017^22^	USA	Method development	NA	NR and/or NA	NA	NR and/or NA
Bennett et al., 2017^30^	USA	Mixed methods research (part of guideline)	Kaiser Permanente Integrated Cardiovascular Health (for-profit)	Multiple conditions	National	21–30
Brouwers et al., 2017^42^	Canada	Mixed methods research (patient engagement in guidelines)	NA	Neoplasms	NA	41–50
Coombs et al., 2017^55^	USA	Qualitative research (part of guideline)	American College of Critical Care (professional)	Patient care	National	21–30
Davidson et al., 2017^60^	USA	Guideline development	NR	Patient care	NR	21–30
Goodman et al., 2017^95^	USA	Guideline development	Multiple organizations (professional)	Diseases of the musculoskeletal system or connective tissue	National	11–20
Goodman et al., 2017^94^; see also Goodman et al., 2017^95^	USA	Guideline development	Multiple organizations (professional)	Diseases of the musculoskeletal system or connective tissue	National	11–20
Gupta et al., 2017^102^	Japan, USA	Guideline development	Multiple organizations (professional)	Diseases of the respiratory system	Multicountry	NR and/or NA
Lambert et al., 2017^142^	Australia, New Zealand	Guideline development	Royal Australian and New Zealand College of Psychiatrists (professional)	Mental, behavioural or neurodevelopmental disorders	Multicountry	41–50
Lennaerts et al., 2017^151^	Netherlands (Kingdom of the)	Guideline development	Dutch Society of Parkinsons Disease Nurse Specialists and ParkinsonNet (professional)	Diseases of the nervous system	National	2–10
Li et al., 2017^153^	China	Guideline development	Fudan University (academic)	Neoplasms	National	NR and/or NA
Masilo & Davhana-Maselesele, 2017^160^	South Africa	Guideline development	NR	Mental, behavioural or neurodevelopmental disorders	National	> 100
Miller et al., 2017^166^	Australia	Guideline development	Kidney Health Australia (nonprofit)	Certain infectious or parasitic diseases	National	11–20
Okely et al., 2017^181^	Australia	Guideline adaptation and/or translation	Australian Government Department of Health and Ageing (government body)	Prevention	National	21–30
Selva et al., 2017^217^	Australia, Belgium, Canada, Colombia, Estonia, France, Germany, Italy, Netherlands (Kingdom of the), New Zealand, Peru, Spain, Switzerland, United Kingdom, USA	Review	NR	NR and/or NA	Multicountry	NR and/or NA
Tremblay et al., 2017^244^	Canada	Guideline development	Canadian Society for Exercise Physiology (professional)	Prevention	National	NR and/or NA
Wiles et al., 2017^267^	Australia	Protocol	NR	Multiple conditions	Facility	NR and/or NA
Brouwers et al., 2018^43^	Canada	Method development	NA	Neoplasms	NA	11–20
Cools et al., 2018^54^	Including Austria, Belgium, Denmark, Estonia, Finland, France, Netherlands (Kingdom of the), Poland (other countries NR)	Guideline development	European Cooperation in Science and Technology Action Differences of Sex Development Network (nonprofit)	Developmental anomalies	Multicountry	NR and/or NA
Davenport et al., 2018^59^	Canada	Guideline development	NR	Prevention	National	> 100
Duff et al., 2018^74^	Australia, New Zealand	Protocol	Kidney Health Australia (nonprofit)	Diseases of the genitourinary system	Multicountry	NR and/or NA
Gibb et al., 2018^92^	Canada, China, Sweden, United Kingdom, USA	Protocol	International ERAS (Enhanced Recovery After Surgery) Society (professional)	Diseases of the digestive system	Multicountry	NR and/or NA
Gibson et al., 2018^93^	Canada	Qualitative research (part of guideline)	NR	Diseases of the nervous system	National	51–100
Grant et al., 2018^99^	Canada, USA	Methodology	Multiple organizations (nonprofit)	Diseases of the nervous system	Multicountry	NR and/or NA
Groenen et al., 2018^101^	Netherlands (Kingdom of the)	Guideline development	Netherlands (Kingdom of the) Comprehensive Cancer Organisation (nonprofit)	Neoplasms	National	2–10
Martin Ginis et al., 2018^159^	Canada	Guideline development	NR	Injury, poisoning or certain other consequences of external causes	National	41–50
Misso et al., 2018^168^	Australia, Netherlands (Kingdom of the), United Kingdom, USA	Guideline development	Multiple organizations (collaboration of various types of organizations)	Endocrine, nutritional or metabolic diseases	Multicountry	> 100
Mottola et al., 2018^175^	Canada	Guideline development	Society of Obstetricians and Gynaecologists of Canada (professional)	Prevention	National	2–10
Rosenberg et al., 2018^207^	USA	Guideline development	United States Department of Veterans Affairs and Department of Defense (government body)	Patient care	National	NR and/or NA
Stephens et al., 2018^225^	United Kingdom	Guideline development	Tissue Viability Society (professional)	Diseases of the skin	National	NR and/or NA
Teede et al., 2018^235^	Australia	Guideline development	Multiple organizations (collaboration of various types of organizations)	Endocrine, nutritional or metabolic diseases	Multicountry	> 100
Tromp et al., 2018^248^	Indonesia	Guideline adaptation and/or translation	Multiple organizations (collaboration of various types of organizations)	Certain infectious or parasitic diseases	National	2–10
Zuckerbrot et al., 2018^276^	USA	Guideline development	American Academy of Pediatrics (professional)	Mental, behavioural or neurodevelopmental disorders	National	NR and/or NA
Angeles-Han et al., 2019^17^	USA	Guideline development	American College of Rheumatology (professional)	Diseases of the musculoskeletal system or connective tissue	National	11–20
Daraz et al., 2019^58^	USA	Methodology	American Society of Hematology (professional)	Diseases of the blood or blood-forming organs	National	21–30
Gutman et al., 2019^103^	Australia	Guideline development	Kidney Health Australia (nonprofit)	Diseases of the genitourinary system	National	11–20
Jardine et al., 2019^119^; see also Miller et al., 2017^166^	Australia	Guideline development	Kidney Health Australia (nonprofit)	Certain infectious or parasitic diseases	National	11–20
Khodyakov et al., 2019^131^	Canada, USA	Guideline development	Multiple organizations (nonprofit)	Diseases of the nervous system	Multicountry	> 100
Kottner et al., 2019^139^	Australia; Belgium; China; China, Hong Kong SAR; France; Germany; Ireland; New Zealand; Singapore; United Kingdom; USA	Protocol	Multiple organizations (professional)	Diseases of the skin	Multicountry	NR and/or NA
Köpke et al., 2019^138^	Bulgaria, Denmark, Germany, Israel, Italy, Netherlands (Kingdom of the), Serbia, Spain, United Kingdom	Guideline development	European Academy of Neurology (professional)	Diseases of the nervous system	Multicountry	> 100
Lam et al., 2019^141^	Belgium, Canada, France, Germany, Greece, Italy, Netherlands (Kingdom of the), Spain, Sweden, Switzerland, United Kingdom, USA	Guideline development	Multiple organizations (professional)	Neoplasms	Multicountry	31–40
Le Roux et al., 2019^146^	France	Guideline development	NR	Multiple conditions	National	21–30
Pangarkar et al., 2019^189^	USA	Guideline development	United States Department of Veterans Affairs and Department of Defense (government body)	Diseases of the musculoskeletal system or connective tissue	National	NR and/or NA
Ramos et al., 2019^204^	USA	Guideline development	Cystic Fibrosis Foundation (nonprofit)	Diseases of the respiratory system	National	2–10
Ringold et al., 2019^205^	USA	Guideline development	American College of Rheumatology (professional)	Diseases of the musculoskeletal system or connective tissue	National	11–20
Sall et al., 2019^209^	USA	Guideline development	United States Department of Veterans Affairs and Department of Defense (government body)	Diseases of the nervous system	National	NR and/or NA
Singh et al., 2019^220^	USA	Guideline development	Multiple organizations (professional)	Diseases of the musculoskeletal system or connective tissue	National	11–20
Zhang et al., 2019^275^	China	Protocol	Guangzhou University of Chinese Medicine (academic)	Diseases of the nervous system	National	NR and/or NA
Armstrong et al., 2020^20^	USA	Guideline development	American Academy of Neurology (professional)	Mental, behavioural or neurodevelopmental disorders	National	> 100
Baldwin et al., 2020^26^	Australia	Guideline development	NR	Multiple conditions	NR	11–20
Bergman et al., 2020^31^	USA	Guideline development	NR	Patient care	Facility	2–10
Blackwood et al., 2020^36^	Australia, Austria, Belgium, Brazil, Canada, Colombia, Finland, France, Germany, Ireland, Luxembourg, Malaysia, Netherlands (Kingdom of the), Spain, Sweden, Ukraine, United Kingdom, USA	Quantitative research (patient engagement in guidelines)	NA	NR and/or NA	NA	NR and/or NA
Chen et al., 2020^49^	China	Guideline development	Chinese Society of Oral Medicine (professional)	Diseases of the digestive system	National	2–10
Doyle, 2020^72^	Australia	Guideline development	Sunshine Coast Hospital and Health Service (hospital)	Mental, behavioural or neurodevelopmental disorders	Facility	NR and/or NA
FitzGerald et al., 2020^82^	USA	Guideline development	American College of Rheumatology (professional)	Diseases of the musculoskeletal system or connective tissue	National	2–10
Hartney et al., 2020^111^	Canada	Guideline development	NR	Mental, behavioural or neurodevelopmental disorders	National	2–10
Hempstead et al., 2020^113^	USA	Commentary	Cystic Fibrosis Foundation (nonprofit)	Diseases of the respiratory system	National	NR and/or NA
Kalot et al., 2020^125^	Canada, USA	Guideline development	Multiple organizations (collaboration of various types of organizations)	Diseases of the blood or blood-forming organs	Multicountry	> 100
Kampling et al., 2020^127^	Germany, Switzerland	Guideline development	NR	Diseases of the nervous system	Multicountry	2–10
Karpusheff et al., 2020^128^	United Kingdom	Commentary	National Institute of Health and Care Excellence (government body)	Mental, behavioural or neurodevelopmental disorders	National	2–10
Khodyakov et al., 2020^132^; see also Khodyakov et al., 2019^131^	Canada, USA	Guideline development	Multiple organizations (nonprofit)	Diseases of the nervous system	Multicountry	> 100
Kim et al., 2020^133^	Australia, Canada, Finland, Netherlands (Kingdom of the), Spain, United Kingdom, USA	Review	Guideline International Network Patient and Public Involvement Working Group (nonprofit)	NR and/or NA	NR	NR and/or NA
Kolasinski et al., 2020^137^	USA	Guideline development	Multiple organizations (professional)	Diseases of the musculoskeletal system or connective tissue	National	NR and/or NA
Lea et al., 2020^147^	United Kingdom	Guideline development	NR	Neoplasms	National	11–20
McMaster et al., 2020^164^	Australia	Guideline development	NR	Mental, behavioural or neurodevelopmental disorders	National	51–100
Morin et al., 2020^173^	Canada	Quantitative research (part of guideline)	Osteoporosis Canada (nonprofit)	Diseases of the musculoskeletal system or connective tissue	National	> 100
Perkins et al., 2020^190^	USA	Guideline development	American Society for Colposcopy and Cervical Pathology	Neoplasms	National	> 100
Petkovic et al., 2020^192^	Australia, Brazil, Canada, Germany, Italy, Lebanon, Netherlands (Kingdom of the), Philippines, Switzerland, United Kingdom, USA	Protocol	Multi-Stakeholder Engagement (MuSE) Consortium (research or guideline consortium)	Other	Multicountry	NR and/or NA
Pomey et al., 2020^197^	Canada	Guideline development	Ministry of Health in Quebec (government body)	Certain infectious or parasitic diseases	National	2–10
Salarvand et al., 2020^208^	Iran (Islamic Republic of)	Guideline adaptation and/or translation	NR	Diseases of the digestive system	National	NR and/or NA
Sammaritano et al., 2020^210^	USA	Guideline development	American College of Rheumatology (professional)	Multiple conditions	National	11–20
Singh et al., 2020^221^	USA	Guideline development	American College of Rheumatology (professional)	Diseases of the musculoskeletal system or connective tissue	National	2–10
Smith et al., 2020^222^	USA	Qualitative research (part of guideline)	Cystic Fibrosis Foundation (nonprofit)	Diseases of the respiratory system	National	2–10
Stephenson et al., 2020^226^	United Kingdom	Guideline development	NR	Mental, behavioural or neurodevelopmental disorders	National	11–20
Townend et al., 2020^242^	43 countries (NR)	Guideline development	Maastricht University (academic)	Developmental anomalies	Global	> 100
van Doormaal et al., 2020^71^	Netherlands (Kingdom of the)	Guideline development	Royal Dutch Society for Physical Therapy (professional)	Diseases of the musculoskeletal system or connective tissue	National	NR and/or NA
Wu et al., 2020^271^	China	Protocol	Jiangxi University of Traditional Chinese Medicine (academic)	Diseases of the musculoskeletal system or connective tissue	National	NR and/or NA
Augustine et al., 2021^25^	Argentina, Australia, Finland, Germany, Italy, New Zealand, Türkiye, USA	Guideline development	Taylor’s Tale (patient advocacy group)	Endocrine, nutritional or metabolic diseases	Multicountry	31–40
Birgisdóttir et al., 2021^34^	Sweden	Guideline development	Swedish National Board of Health and Welfare (government body)	Patient care	National	> 100
Björkqvist et al., 2021^35^	Multiple European countries (NR)	Methodology	European Association of Urology (professional)	Neoplasms	Multicountry	NR and/or NA
Bulley et al., 2021^45^	United Kingdom	Mixed methods research (part of guideline)	NHS (government body)	Diseases of the nervous system	National	2–10
Courbiere et al., 2021^56^	France	Guideline development	NR	Diseases of the genitourinary system	National	2–10
Fauci et al., 2021^79^	Italy	Guideline adaptation and/or translation	Italian National Institute of Health (government body)	Injury, poisoning or certain other consequences of external causes	National	NR and/or NA
Fraenkel et al., 2021^85^; see also Fraenkel et al., 2016^86^	USA	Guideline development	American College of Rheumatology (professional)	Diseases of the musculoskeletal system or connective tissue	National	11–20
Grant et al., 2021^98^; see also Grant et al., 2018^99^	Canada, USA	Methodology	Multiple organizations (nonprofit)	Diseases of the nervous system	Multicountry	NR and/or NA
Hua et al., 2021^78^	China	Protocol	Jiangxi University of Traditional Chinese Medicine (academic)	Diseases of the musculoskeletal system or connective tissue	National	NR and/or NA
Kamaruzaman et al., 2021^126^	Malaysia	Guideline development	Malaysian Health Technology Assessment Section at Ministry of Health (government body)	Multiple conditions	National	NR and/or NA
Maz et al., 2021^162^	USA	Guideline development	Multiple organizations (professional)	Diseases of the immune system	National	11–20
Piggott et al., 2021^194^	Global (NR)	Guideline development	Multiple Sclerosis International Federation (nonprofit)	Diseases of the nervous system	Global	NR and/or NA
Sawyer et al., 2021^213^	Australia	Guideline development	NR	External causes of morbidity or mortality	National	2–10
Tendal et al., 2021^237^	Australia	Guideline development	Australian National COVID-19 (coronavirus disease) Clinical Evidence Taskforce (government body)	Certain infectious or parasitic diseases	National	2–10
Volerman et al., 2021^258^	USA	Guideline development	Multiple organizations (professional)	Diseases of the respiratory system	National	NR and/or NA
Werner et al., 2021^263^	Germany	Quantitative research (part of guideline)	Charité Universitätsmedizin Berlin (academic)	Neoplasms	National	31–40
Assmann et al., 2022^23^	Czechia, Greece, Ireland, Italy, Lithuania, Netherlands (Kingdom of the), Norway, Switzerland, United Kingdom	Guideline development	Multiple organizations (professional)	Diseases of the digestive system	Multicountry	2–10
Biggane et al., 2022^33^	United Kingdom	Qualitative research (patient engagement in guidelines)	National Institute of Health and Care Excellence (government body)	Multiple conditions	National	NR and/or NA
Clancy et al., 2022^51^	Australia	Guideline development	Australian Meals on Wheels Association (nonprofit)	Prevention	National	> 100
Ferguson et al., 2022^81^	Canada	Guideline development	Canadian Research Initiative in Substance Misuse (research or guideline consortium)	Mental, behavioural or neurodevelopmental disorders	National	2–10
Francisco et al., 2022^87^	Argentina, Armenia, Australia, Belgium, Brazil, Canada, Colombia, Cyprus, Estonia, Finland, France, Georgia, Germany, Iran (Islamic Republic of), Italy, Netherlands (Kingdom of the), New Zealand, Poland, Portugal, South Africa, Spain, Sweden, Switzerland, United Kingdom, USA	Guideline development	NR	Endocrine, nutritional or metabolic diseases	Multicountry	> 100
Gehring et al., 2022^91^	Canada	Quantitative research (part of guideline)	Obesity Canada (nonprofit)	Endocrine, nutritional or metabolic diseases	National	21–30
Gorelik et al., 2022^96^	USA	Guideline development	Multiple organizations (professional)	Diseases of the immune system	National	11–20
Haesler et al., 2022^105^	Australia; China, Hong Kong SAR; France; Germany; New Zealand; Singapore; USA	Quantitative research (part of guideline)	Multiple organizations (professional)	Diseases of the skin	Multicountry	> 100
Littlejohn et al., 2022^156^	Australia, Canada, Cyprus, France, Greece, Ireland, United Kingdom, USA	Guideline development	NR	Mental, behavioural or neurodevelopmental disorders	Multicountry	21–30
Mager et al., 2022^158^	Canada	Guideline development	NR	Diseases of the digestive system	National	> 100
Montero-Odasso et al., 2022^171^	Argentina, Australia, Bolivia (Plurinational State of), Brazil, Canada, Chile, China, Colombia, Costa Rica, Denmark, Ecuador, Finland, France, Germany, Iran (Islamic Republic of), Ireland, Israel, Italy, Japan, Malaysia, Mexico, Netherlands (Kingdom of the), New Zealand, Panama, Paraguay, Peru, Qatar, Republic of Korea, Singapore, South Africa, Spain, Sweden, Switzerland, Türkiye, Uganda, United Kingdom, USA, Uruguay,	Guideline development	British Geriatrics Society (professional)	Injury, poisoning or certain other consequences of external causes	Multicountry	2–10
Moore et al., 2022^172^	Canada	Quantitative research (patient engagement in guidelines)	Canadian Task Force on Preventive Health Care (government body)	Other	National	NR and/or NA
Okely et al., 2022^182^	Australia	Guideline adaptation and/or translation	Australian Government Department of Health and Ageing (government body)	Prevention	National	NR and/or NA
Onel et al., 2022^183^	USA	Guideline development	Multiple organizations (professional)	Diseases of the musculoskeletal system or connective tissue	National	11–20
Ralph et al., 2022^203^	Australia	Guideline development	National Eating Disorders Collaboration (research or guideline consortium)	Mental, behavioural or neurodevelopmental disorders	National	NR and/or NA
Saunders et al., 2022^212^	Canada	Guideline development	Sedentary Behaviour Research Network (nonprofit)	Prevention	National	21–30
Song et al., 2022^224^	China	Guideline development	Chinese Pharmacological (professional)Society	Neoplasms	National	> 100
Sweeney et al., 2022^229^	United Kingdom	Guideline development	NR	Mental, behavioural or neurodevelopmental disorders	National	51–100
Synnot et al., 2022^232^	Australia, United Kingdom, USA, Europe (NR), and other (NR)	Review	NR	NR and/or NA	Multicountry	NR and/or NA
Warrier et al., 2022^261^	India	Guideline development	NR	Diseases of the nervous system	National	11–20
Abel et al., 2023^12^	Switzerland	Guideline development	NR	Multiple conditions	National	41–50
Adams et al., 2023^13^	Australia	Guideline development	NR	Mental, behavioural or neurodevelopmental disorders	Facility	NR and/or NA
Andreasen et al., 2023^16^	Denmark, Finland, Iceland, Norway, Sweden	Guideline development	Clinical Practice Committee of the Scandinavian Society of Anaesthesiology and Intensive care medicine (professional)	Diseases of the circulatory system	Multicountry	2–10
Atkins et al., 2023^24^	Australia	Guideline development	National Heart Foundation of Australia (nonprofit)	Diseases of the circulatory system	National	31–40
Bass et al., 2023^27^	USA	Guideline development	American College of Rheumatology (professional)	Diseases of the musculoskeletal system or connective tissue	National	2–10
Bray et al., 2023^41^	Australia, Brazil, Cambodia, Canada, Indonesia, Ireland, Jordan, Malawi, Netherlands (Kingdom of the), New Zealand, Norway, South Africa, Spain, Sweden, United Kingdom, USA, Zambia	Guideline development	ISupport international collaboration (international organization)	Patient care	Global	> 100
Caoili et al., 2023^48^	USA	Guideline development	NR	Mental, behavioural or neurodevelopmental disorders	NR	11–20
Chung et al., 2023^50^	Argentina, Australia, Belgium, Brazil, Canada, China, Dominican Republic, France, India, Japan, Netherlands (Kingdom of the), Poland, Republic of Korea, Singapore, USA	Guideline development	Heart Rhythm Society and the Latin American Heart Rhythm Society, in collaboration with the American College of Cardiology, American Heart Association, Pediatric and Congenital Electrophysiology Society, International Society of Holter and Noninvasive Electrocardiology, and Heart Failure Society of America (professional)	Diseases of the circulatory system	Global	NR and/or NA
Dawson et al., 2023^61^	USA	Guideline development	United States Food and Drug Administration (government body)	Diseases of the digestive system	National	51–100
De Cuyper et al., 2023^62^	Belgium	Guideline development	University of Leuven (academic)	Mental, behavioural or neurodevelopmental disorders	National	21–30
Demers et al., 2023^64^	Canada	Guideline development	Canadian Physiotherapy Association (professional)	Mental, behavioural or neurodevelopmental disorders	National	11–20
Demetri et al., 2023^65^	United Kingdom	Protocol	Multiple organizations (collaboration of various types of organizations)	Patient care	National	> 100
Geberhiwot et al., 2023^90^	United Kingdom	Guideline development	NHS (government body)	Endocrine, nutritional or metabolic diseases	National	NR and/or NA
Haesler et al., 2023^104^	Australia; China, Hong Kong SAR; New Zealand; Singapore	Quantitative research (part of guideline)	Multiple organizations (professional)	Diseases of the circulatory system	Multicountry	21–30
Hannon et al., 2023^107^	USA	Guideline development	American College of Rheumatology (professional)	Diseases of the musculoskeletal system or connective tissue	National	2–10
Kabir et al., 2023^124^	United Kingdom	Guideline development	DECIDE (Delphi Expert Consensus Statement on Inflammatory Bowel Disease Dysplasia Shared Management E-Decision-Making) steering group (University College London Hospitals NHS Foundation Trust, Imperial College London, Nottingham University Hospitals NHS Trust, Royal Free Hospital, St Mark’s Hospital, IRCCS [Scientific Institute for Research, Hospitalization and Healthcare] Humanitas Research Hospital, University Hospital Leuven, British Society of Gastroenterology, European Crohn’s and Colitis Organisation, Leeds Teaching Hospitals NHS Trust, Guts United Kingdom, Dr Falk Pharma; collaboration of various types of organizations)	Diseases of the digestive system	National	> 100
Lenet et al., 2023^150^	Canada	Guideline development	Ottawa Consensus on Intraoperative Patient Blood Management (collaboration of various types of organizations)	Diseases of the blood or blood-forming organs	National	11–20
Liy-Wong et al., 2023^157^	Canada	Guideline development	NR	Diseases of the blood or blood-forming organs	National	11–20
Mayer et al., 2023^161^	Canada	Guideline development	NR	Mental, behavioural or neurodevelopmental disorders	National	2–10
McArthur et al., 2023^163^	Canada, Netherlands (Kingdom of the), United Kingdom	Review	NR	Mental, behavioural or neurodevelopmental disorders	Multicountry	NR and/or NA
Mendes et al., 2023^165^	Brazil	Qualitative research (part of guideline)	NR	Diseases of the blood or blood-forming organs	NR	11–20
Mochamat et al., 2023^169^	Germany	Guideline development	Department of Palliative Care of the University Hospital of Bonn (academic)	Patient care	Facility	2–10
Morton et al., 2023^174^	Australia, Belgium, Canada, Denmark, France, Germany, Italy, Netherlands (Kingdom of the), Spain, Switzerland, United Kingdom	Guideline development	Galderma (for-profit)	Diseases of the skin	Multicountry	NR and/or NA
Mulcahy et al., 2023^176^	Australia	Guideline development	NR	Mental, behavioural or neurodevelopmental disorders	National	11–20
Nunnerley et al., 2023^178^	Australia, New Zealand	Qualitative research (part of guideline)	Australian and New Zealand Spinal Cord Injury physiotherapists (research or guideline consortium)	Injury, poisoning or certain other consequences of external causes	Multicountry	21–30
Ostgathe et al., 2023^185^	Germany	Guideline development	Multiple organizations (research or guideline consortium)	Patient care	National	2–10
Persaud et al., 2023^191^	Canada	Guideline development	Equitable Preventive Praxis Initiative in Canada (collaboration of various types of organizations)	Multiple conditions	National	NR and/or NA
Petkovic et al., 2023^2^	Canada, Croatia, Denmark, Germany, India, Italy, Lebanon, Nigeria, Qatar, South Africa, Spain, Tunisia, United Kingdom, USA	Methodology	Multi-Stakeholder Engagement (MuSE) Consortium (collaboration of various types of organizations)	Multiple conditions	Global	NR and/or NA
Popenhagen et al., 2023^198^	Argentina, Australia, Austria, Brazil, Croatia, Ireland, Mexico, New Zealand, Pakistan, South Africa, Spain, United Kingdom, USA	Guideline development	Debra International (nonprofit)	Diseases of the skin	Multicountry	41–50
Raaijmakers et al., 2023^202^	Netherlands (Kingdom of the)	Mixed methods research (part of guideline)	NR	Patient care	National	NR and/or NA
Sweegers et al., 2023^228^	Netherlands (Kingdom of the)	Guideline development	Netherlands Organisation for Health Research and Development (ZonMW; government body)	Neoplasms	National	NR and/or NA
Synnot et al., 2023^231^	Australia, New Zealand	Guideline development	Multiple organizations (research or guideline consortium)	Multiple conditions	Multicountry	NR and/or NA
Teede et al., 2023^236^	Australia; Brazil; Canada; China; China, Hong Kong SAR; Finland; Georgia; India; Iran (Islamic Republic of); Ireland; Italy; Netherlands (Kingdom of the); Norway; Serbia; Sri Lanka; South Africa; Sweden; Türkiye; United Kingdom; USA; others (NR)	Guideline development	Australian Government via the National Health Medical Research Council, supported by American Society for Reproductive Medicine, Endocrine Society, European Society for Human Reproduction and Embryology, and European Society for Endocrinology (collaboration of various types of organizations)	Endocrine, nutritional or metabolic diseases	Global	> 100
Thériault et al., 2023^239^	Canada	Guideline development	Canadian Task Force on Preventive Health Care (government body)	Injury, poisoning or certain other consequences of external causes	National	31–40
Tronco Hernandez et al., 2023^249^	United Kingdom	Guideline development	NR	Certain infectious or parasitic diseases	National	11–20
van Teunenbroek et al., 2023^254^	Netherlands (Kingdom of the)	Guideline development	Netherlands Comprehensive Cancer Organisation (nonprofit)	Patient care	National	21–50
Wabnitz et al., 2023^259^	Germany	Qualitative research (part of guideline)	Association of the Scientific Medical Societies (Arbeitsgemeinschaft für Medizinisch-Wissenschaftliche Fachgesellschaften; professional)	Certain infectious or parasitic diseases	National	2–10
Wood et al., 2023^269^	Canada	Guideline development	Canadian Research Initiative in Substance Misuse (collaboration of various types of organizations)	Mental, behavioural or neurodevelopmental disorders	National	11–20
Zeng et al., 2023^274^	NR (multiple)	Methodology	British Medical Journal Rapid Recommendations (international organization)	NR and/or NA	Global	NR and/or NA
Alteri et al., 2024^15^	Argentina, Austria, Barbados, Belgium, Bulgaria, Croatia, Denmark, Finland, Germany, Italy, Netherlands (Kingdom of the), Portugal, Romania, Spain, Sweden, Switzerland, United Arab Emirates, United Kingdom, USA, Yemen	Guideline development	European Society of Human Reproduction and Embryology (professional)	Diseases of the genitourinary system	Multicountry	> 100
Apeldoorn et al., 2024^18^	Netherlands (Kingdom of the)	Guideline development	Royal Dutch Society for Physical Therapy (professional)	Diseases of the musculoskeletal system or connective tissue	National	2–10
Bennett et al., 2024^29^	Australia	Guideline development	Audiology Australia, Australian Government’s Department of Health (collaboration of various types of organizations)	Diseases of the ear or mastoid process	National	NR and/or NA
Blokzijl et al., 2024^37^	Netherlands (Kingdom of the)	Guideline development	NR	Diseases of the blood or blood-forming organs	National	NR and/or NA
Bulley et al., 2024^44^	United Kingdom	Guideline development	Association of Chartered Physiotherapists in Neurology in the United Kingdom (professional)	Diseases of the nervous system	National	11–20
Burton et al., 2024^46^	United Kingdom	Guideline development	United Kingdom Kidney Association (professional)	Diseases of the genitourinary system	National	51–100
Clinical Guideline Committee et al., 2024^238^	USA	Guideline development	American Society of Addiction Medicine and American Academy of Addiction Psychiatry (professional)	Mental, behavioural or neurodevelopmental disorders	National	NR and/or NA
Cook et al., 2024^53^	United Kingdom	Guideline development	NHS; University of Southampton, Hepatitis C Trust, University Hospitals Birmingham NHS Trust, University of Birmingham, NHS Tayside, University of Dundee, Newcastle University, Newcastle-upon-Tyne Hospitals NHS Foundation Trust, Community Pharmacy South Central, University Hospitals Southampton, Carter’s Chemist (South Shields), Community Pharmacy Tees Valley, Community Pharmacy Surrey and Sussex, King’s College Hospital, Royal Cornwall Hospital, St George’s NHS Foundation Trust, University Hospitals of Leicester, University of Manchester, Manchester University NHS Foundation Trust, North Manchester General Hospital, University Hospital of Wales (collaboration of various types of organizations)	Certain infectious or parasitic diseases	National	NR and/or NA
Curtis et al., 2024^57^	USA	Guideline development	Centers for Disease Control and Prevention (government body)	Prevention	National	51–100
Deschmann et al., 2024^69^	Canada, France, Italy, Netherlands (Kingdom of the), Norway, Spain, United Kingdom, USA	Guideline development	Neonatal Transfusion Network in Europe and the USA (professional)	Certain conditions originating in the perinatal period	Global	NR and/or NA
du Plessis et al., 2024^73^	South Africa	Qualitative research (part of guideline)	NR	Mental, behavioural or neurodevelopmental disorders	National	11–20
Fautrel et al., 2024^80^	France	Guideline development	French Society of Rheumatology (professional)	Diseases of the musculoskeletal system or connective tissue	National	11–20
Green et al., 2024^100^	USA	Guideline development	Cystic Fibrosis Foundation (nonprofit)	Endocrine, nutritional or metabolic diseases	National	NR and/or NA
Harmsen et al., 2024^109^	Netherlands (Kingdom of the)	Methodology	Netherlands Organisation for Health Research and Development (government body)	Multiple conditions	National	NR and/or NA
Hart et al., 2024^110^	NR (33 countries)	Guideline development	Multinational Association of Supportive Care in Cancer and American Society of Clinical Oncology (professional)	Neoplasms	Global	11–20
Heran et al., 2024^114^	China	Guideline development	Heart and Stroke Foundation of Canada (nonprofit)	Diseases of the circulatory system	National	11–20
Hosie et al., 2024^117^	United Kingdom	Guideline development	Scottish Dental Clinical Effectiveness Programme (professional)	Diseases of the digestive system	National	> 100
Johannes et al., 2024^120^	South Africa	Guideline development	NR	Prevention	Facility	> 100
Johnson et al., 2024^122^	USA	Guideline development	American College of Rheumatology, American College of Chest Physicians (professional)	Diseases of the musculoskeletal system or connective tissue	National	21–50
Johnson et al., 2024^123^; see also Johnson et al., 2024^122^	USA	Guideline development	American College of Rheumatology, American College of Chest Physicians(professional)	Diseases of the musculoskeletal system or connective tissue	National	21–50
Liang et al., 2024^154^	China	Guideline development	NR	Diseases of the genitourinary system	National	2–10
Mirza et al., 2024^167^	USA	Guideline development	American College of Rheumatology, American College of Chest Physicians (professional)	Diseases of the respiratory system	National	21–50
Panay et al., 2024^188^	Australia, Canada, Egypt, France, Hungary, India, Malta, Netherlands (Kingdom of the), Poland, Portugal, Russian Federation, United Kingdom, USA	Guideline development	American Society for Reproductive Medicine, European Society of Human Reproduction and Embryology, and International Menopause Society (professional)	Endocrine, nutritional or metabolic diseases	Global	NR and/or NA
Petkovic et al., 2024^193^	NR	Review	NR	NR and/or NA	NR	NR and/or NA
Pilkington et al., 2024^195^	Australia, United Kingdom, USA	Guideline development	Enhanced Recovery After Surgery Society (professional)	Pregnancy, childbirth or the puerperium	Global	2–10
Sarrafzadegan et al., 2024^211^	Iran (Islamic Republic of)	Guideline development	Iranian Ministry of Health (government body)	Diseases of the circulatory system	National	11–20
Scholes-Robertson et al., 2024^215^	Australia, New Zealand	Methodology	Caring for Australians and New Zealanders (professional)	Diseases of the genitourinary system	Multicountry	NR and/or NA
Stubbs et al., 2024^227^	United Kingdom	Guideline development	Improving Care in Elderly Neurosurgery Initiative Group, comprising NHS, University of Cambridge, Cambridge University Hospitals NHS Trust, Oxford University Hospitals NHS Trust, Erasmus Medical Center Rotterdam, NHS Lothian, Norfolk and Norwich University Hospitals NHS Trust, Salford Royal NHS Trust, University Hospital Southampton, Queens Medical Centre Nottingham, The Brain Tumour Charity, Imperial College London, Barts Health NHS Trust, Barking, Havering and Redbridge University Trust, Queen Mary University of London, Manchester University NHS Foundation Trust, Central London Community Healthcare NHS Trust, South West Neurosurgical Centre Plymouth, THIS (The Healthcare Improvement Studies) Institute, North Bristol NHS Trust, Neurological Alliance, King’s College London NHS Foundation Trust, St George’s Hospital London, Nottingham University Hospitals NHS Trust, University of Sheffield, University of Nottingham, EXEP Consulting, Leeds General Infirmary, University College London Hospitals NHS Foundation Trust (collaboration of various types of organizations)	Diseases of the circulatory system	National	NR and/or NA
van Gastel et al., 2024^145^	Austria, Belgium, France, Germany, Ireland, Italy, Netherlands (Kingdom of the), Slovenia, Sweden, Switzerland, United Kingdom, USA	Guideline development	International Shoulder Instability Group (collaboration of various types of organizations)	Injury, poisoning or certain other consequences of external causes	Global	NR and/or NA
van Teunenbroek et al., 2024^255^; see also van Teunenbroek et al., 2023^254^	Netherlands (Kingdom of the)	Guideline development	Netherlands Comprehensive Cancer Organisation (nonprofit)	Patient care	National	21–50
Verstraten-Oudshoorn et al., 2024^257^	Netherlands (Kingdom of the)	Qualitative research (part of guideline)	NR	Developmental anomalies	National	21–50
Wentz et al., 2024^262^	USA	Quantitative research (part of guideline)	NR	Developmental anomalies	National	51–100
Wickremsinhe et al., 2024^265^	United Kingdom	Guideline development	National Institute for Health and Care Research (government body)	Mental, behavioural or neurodevelopmental disorders	National	11–20
Xue et al., 2024^272^	China	Guideline development	Chinese Association of Integrative Medicine, Gansu Province Clinical Research Center of Integrative Anesthesiology, Anesthesia Pain Medical Center of Gansu Provincial Hospital of Traditional Chinese Medicine, WHO Collaborating Center for Guideline Implementation and Knowledge Translation, Chinese Grading of Recommendations, Assessment, Development and Evaluation Center, Gansu Provincial Center for Medical Guideline Industry Technology, Evidence-based Medicine Center of Lanzhou University (collaboration of various types of organizations)	Diseases of the digestive system	National	51–100
Eapen et al., 2025^77^	USA	Guideline development	United States Department of Veterans Affairs and Department of Defense (government body)	Diseases of the circulatory system	National	NR and/or NA
Granja-Dominguez et al., 2025^97^	Multiple European countries (NR)	Methodology	European Reference Networks (professional)	Multiple conditions	Multicountry	NR and/or NA
Herbst et al., 2025^115^	Germany	Guideline development	Hannover Medical School (academic)	Patient care	National	NR and/or NA
Johnson et al., 2025^121^	Australia	Guideline development	University of Melbourne, Royal Children’s Hospital, Mental Health First Aid, La Trobe University (collaboration of various types of organizations)	Mental, behavioural or neurodevelopmental disorders	National	21–50
Klein Haneveld et al., 2025^135^	16 European countries (NR)	Qualitative research (patient engagement in guidelines)	European Reference Network ITHACA (Intellectual disability, TeleHealth, Autism and Congenital Anomalies; nonprofit)	Developmental anomalies	Multicountry	NR and/or NA
Narula et al., 2025^177^	USA	Methodology	Society of American Gastrointestinal and Endoscopic Surgeons (professional)	Diseases of the digestive system	National	NR and/or NA
Ostacher et al., 2025^184^	USA	Guideline development	United States Department of Veterans Affairs and Department of Defense (government body)	Mental, behavioural or neurodevelopmental disorders	National	NR and/or NA
Palomba et al., 2025^187^	Italy	Guideline development	Italian Society of Human Reproduction and the Italian Centers for the Study and Conservation of Eggs and Sperm (professional)	Diseases of the genitourinary system	National	NR and/or NA
Qaseem et al., 2025^200^	USA	Guideline development	American College of Physicians (professional)	Diseases of the nervous system	National	2–10
Quesada et al., 2025^201^	31 countries (NR)	Guideline development	European Society of Pathology, French Society of Predictive and Personalized Medicine, Groupe d’Investigateurs Nationaux pour l’Etude des cancers de l’ovaire et du sein, and Cours St Paul (professional)	Neoplasms	Global	2–10
Soegaard et al., 2025^223^	Denmark	Guideline development	National Pressure Ulcer Alliance (nonprofit)	Injury, poisoning or certain other consequences of external causes	National	2–10
Synnot et al., 2025^230^	Australia, Cameroon, Canada, Egypt, Ghana, Iraq, Namibia, Pakistan, Syrian Arab Republic, United Kingdom	Qualitative research (patient engagement in guidelines)	NA	Multiple conditions	NA	21–50
van Cooten et al., 2025^251^	Netherlands (Kingdom of the)	Guideline development	Multiple organizations (collaboration of various types of organizations)	Multiple conditions	National	NR and/or NA
Vella et al., 2025^256^	Australia	Guideline development	Multiple organizations (collaboration of various types of organizations)	Mental, behavioural or neurodevelopmental disorders	National	21–50
Wang et al., 2025^260^	China	Guideline development	Refractive Surgery Group of Chinese Ophthalmologist Association, International Society of Refractive Surgery, WHO Collaborating Centre for Guideline Implementation and Knowledge Translation, Lanzhou University Grading of Recommendations Assessment, Development, and Evaluation Center (professional)	Diseases of the visual system	National	> 100
Yang et al., 2025^273^	China	Guideline development	NR	Diseases of the circulatory system	National	> 100

ICD-11: International Statistical Classification of Diseases and Related Health Problems, 11th revision; NHS: United Kingdom National Health Service; NA: not applicable; NR: not reported; SAR: Special Administrative Region; WHO: World Health Organization.

### Characteristics of reviewed publications

We present the characteristics of included publications in Table 1, and a summary of these in Box 1 (more detailed information available in the online repository).^11^ Most unique studies described guideline development (182/258; 70.5%) or primary research conducted for guideline development (27; 10.5%).

Box 1 **Characteristics of 258 studies^a^ identified in a methodological review of community engagement in guideline development**

**Type of study**

- Guideline development: 182 studies^12^^–^^18^^,^^20^^,^^23^^–^^29^^,^^31^^,^^32^^,^^34^^,^^37^^–^^41^^,^^44^^,^^46^^–^^51^^,^^53^^,^^54^^,^^56^^,^^57^^,^^59^^–^^64^^,^^66^^–^^72^^,^^75^^–^^77^^,^^80^^–^^82^^,^^84^^–^^88^^,^^90^^,^^94^^–^^96^^,^^100^^–^^103^^,^^107^^,^^110^^–^^112^^,^^114^^,^^115^^,^^117^^–^^127^^,^^129^^,^^131^^,^^132^^,^^134^^,^^136^^–^^138^^,^^141^^–^^147^^,^^150^^–^^154^^,^^156^^–^^162^^,^^164^^,^^166^^–^^171^^,^^174^^–^^176^^,^^183^^–^^191^^,^^194^^–^^201^^,^^203^^–^^205^^,^^207^^,^^209^^–^^214^^,^^218^^–^^221^^,^^223^^–^^229^^,^^231^^,^^233^^–^^240^^,^^242^^–^^247^^,^^249^^,^^251^^,^^253^^–^^256^^,^^258^^,^^260^^,^^261^^,^^265^^,^^266^^,^^268^^–^^273^^,^^276^^,^^277^
- Primary research for guideline development: 27 studies^30^^,^^45^^,^^55^^,^^73^^,^^83^^,^^89^^,^^91^^,^^93^^,^^104^^,^^105^^,^^116^^,^^148^^,^^155^^,^^165^^,^^173^^,^^178^^–^^180^^,^^202^^,^^222^^,^^241^^,^^250^^,^^257^^,^^259^^,^^262^^–^^264^
- Method: 14 studies^2^^,^^22^^,^^35^^,^^43^^,^^52^^,^^58^^,^^97^^–^^99^^,^^109^^,^^130^^,^^140^^,^^177^^,^^215^^,^^274^
- Protocol: nine studies^65^^,^^74^^,^^78^^,^^92^^,^^139^^,^^192^^,^^267^^,^^271^^,^^275^
- Review: nine studies^19^^,^^108^^,^^133^^,^^149^^,^^163^^,^^193^^,^^217^^,^^232^^,^^252^
- Primary research about patient engagement: nine studies^21^^,^^33^^,^^36^^,^^42^^,^^106^^,^^135^^,^^172^^,^^216^^,^^230^
- Guideline adaptation and/or translation: five studies^79^^,^^181^^,^^182^^,^^208^^,^^248^
- Commentary: three studies^113^^,^^128^^,^^206^

**Guideline topic**

- Mental health or neurological conditions: 50 studies^13^^,^^20^^,^^32^^,^^40^^,^^44^^,^^45^^,^^48^^,^^62^^,^^64^^,^^72^^,^^73^^,^^81^^,^^88^^,^^93^^,^^98^^,^^99^^,^^108^^,^^111^^,^^118^^,^^121^^,^^128^^,^^129^^,^^131^^,^^132^^,^^134^^,^^138^^,^^142^^–^^144^^,^^151^^,^^156^^,^^160^^,^^161^^,^^163^^,^^164^^,^^176^^,^^184^^,^^194^^,^^200^^,^^203^^,^^226^^,^^229^^,^^238^^,^^253^^,^^256^^,^^261^^,^^265^^,^^266^^,^^269^^,^^275^^–^^277^
- General patient care and health promotion: 35 studies^31^^,^^34^^,^^41^^,^^51^^,^^55^^,^^57^^,^^59^^,^^60^^,^^65^^,^^83^^,^^89^^,^^112^^,^^115^^,^^116^^,^^120^^,^^148^^,^^169^^,^^170^^,^^175^^,^^180^^–^^182^^,^^185^^,^^202^^,^^207^^,^^212^^,^^219^^,^^233^^,^^243^^–^^247^^,^^254^^,^^255^^,^^268^
- Musculoskeletal, connective tissue or immune conditions: 25 studies^17^^,^^18^^,^^27^^,^^47^^,^^71^^,^^78^^,^^80^^,^^82^^,^^85^^,^^86^^,^^94^^–^^96^^,^^107^^,^^122^^,^^123^^,^^137^^,^^162^^,^^173^^,^^183^^,^^189^^,^^199^^,^^205^^,^^218^^,^^220^^,^^221^^,^^264^^,^^271^
- Circulatory or respiratory conditions: 20 studies^16^^,^^24^^,^^50^^,^^52^^,^^77^^,^^102^^,^^104^^,^^113^^,^^114^^,^^127^^,^^130^^,^^140^^,^^167^^,^^204^^,^^209^^,^^211^^,^^222^^,^^227^^,^^258^^,^^273^
- Cancer: 16 studies^35^^,^^38^^,^^42^^,^^43^^,^^101^^,^^110^^,^^136^^,^^141^^,^^147^^,^^153^^,^^190^^,^^201^^,^^216^^,^^224^^,^^228^^,^^263^
- Genital or urinary system conditions: 14 studies^15^^,^^46^^,^^56^^,^^66^^–^^68^^,^^74^^,^^103^^,^^154^^,^^187^^,^^196^^,^^214^^,^^215^^,^^240^^,^^241^
- Infectious or parasitic diseases: 12 studies^14^^,^^28^^,^^53^^,^^119^^,^^152^^,^^166^^,^^179^^,^^197^^,^^237^^,^^248^^,^^249^^,^^259^^,^^270^
- Injury or violence: 11 studies^63^^,^^79^^,^^84^^,^^145^^,^^155^^,^^159^^,^^171^^,^^178^^,^^213^^,^^223^^,^^239^
- Endocrine, nutritional or metabolic diseases: 10 studies^25^^,^^75^^,^^76^^,^^87^^,^^90^^,^^91^^,^^100^^,^^168^^,^^188^^,^^235^^,^^236^
- Digestive system conditions: 10 studies^23^^,^^49^^,^^61^^,^^92^^,^^117^^,^^124^^,^^158^^,^^177^^,^^208^^,^^272^
- Multiple conditions or not specified: 30 studies^2^^,^^12^^,^^19^^,^^21^^,^^22^^,^^26^^,^^30^^,^^33^^,^^36^^,^^70^^,^^97^^,^^106^^,^^109^^,^^126^^,^^133^^,^^146^^,^^149^^,^^191^^,^^193^^,^^206^^,^^210^^,^^217^^,^^230^^–^^232^^,^^234^^,^^250^^,^^251^^,^^267^^,^^274^
- Other: 25 studies^29^^,^^37^^,^^39^^,^^54^^,^^58^^,^^69^^,^^105^^,^^125^^,^^135^^,^^139^^,^^150^^,^^157^^,^^165^^,^^172^^,^^174^^,^^186^^,^^192^^,^^195^^,^^198^^,^^225^^,^^242^^,^^252^^,^^257^^,^^260^^,^^262^

**Guideline target population*^b^***

- People affected by the guidelines: 242 studies^12^^–^^82^^,^^84^^–^^105^^,^^107^^,^^109^^–^^116^^,^^118^^,^^119^^,^^121^^–^^151^^,^^153^^–^^171^^,^^173^^–^^176^^,^^178^^–^^191^^,^^193^^–^^214^^,^^216^^–^^229^^,^^231^^–^^246^^,^^248^^–^^255^^,^^257^^,^^258^^,^^260^^–^^273^^,^^275^^–^^277^
- Caregivers: 11 studies^31^^,^^32^^,^^34^^,^^100^^,^^115^^,^^118^^,^^160^^,^^170^^,^^250^^,^^266^^,^^268^
- Public or general population: nine studies^39^^,^^83^^,^^117^^,^^120^^,^^152^^,^^192^^,^^247^^,^^256^^,^^259^
- Guideline developers: six studies^2^^,^^172^^,^^177^^,^^215^^,^^230^^,^^274^
- Not specified: two studies^106^^,^^108^

**Type of guideline-issuing entity**

- Professional organization: 73 studies^15^^–^^18^^,^^20^^,^^23^^,^^27^^,^^35^^,^^38^^,^^44^^,^^46^^,^^47^^,^^49^^,^^50^^,^^52^^,^^55^^,^^58^^,^^64^^,^^69^^,^^71^^,^^80^^,^^82^^,^^85^^,^^86^^,^^92^^,^^94^^–^^97^^,^^102^^,^^104^^–^^107^^,^^110^^,^^117^^,^^122^^,^^123^^,^^130^^,^^137^^–^^142^^,^^151^^,^^162^^,^^167^^,^^171^^,^^175^^,^^177^^,^^183^^,^^187^^,^^188^^,^^195^^,^^200^^,^^201^^,^^205^^,^^206^^,^^210^^,^^214^^,^^215^^,^^220^^,^^221^^,^^224^^,^^225^^,^^238^^,^^243^^–^^247^^,^^258^^–^^260^^,^^276^
- Nonprofit organization: 38 studies^24^^,^^32^^,^^39^^,^^40^^,^^51^^,^^54^^,^^74^^,^^84^^,^^91^^,^^98^^–^^103^^,^^108^^,^^113^^,^^114^^,^^119^^,^^129^^,^^131^^–^^135^^,^^143^^,^^144^^,^^149^^,^^166^^,^^173^^,^^186^^,^^194^^,^^198^^,^^204^^,^^212^^,^^216^^,^^222^^,^^223^^,^^233^^,^^240^^,^^241^^,^^254^^,^^255^
- Government body: 29 studies^33^^,^^34^^,^^45^^,^^57^^,^^61^^,^^77^^,^^79^^,^^83^^,^^90^^,^^109^^,^^116^^,^^126^^,^^128^^,^^172^^,^^180^^–^^182^^,^^184^^,^^189^^,^^197^^,^^207^^,^^209^^,^^211^^,^^219^^,^^228^^,^^237^^,^^239^^,^^252^^,^^265^
- University: 11 studies^62^^,^^63^^,^^78^^,^^115^^,^^120^^,^^153^^,^^169^^,^^242^^,^^263^^,^^271^^,^^275^
- Research or guideline consortium: nine studies^81^^,^^88^^,^^112^^,^^178^^,^^185^^,^^192^^,^^203^^,^^231^^,^^234^
- Hospital: five studies^72^^,^^118^^,^^170^^,^^196^^,^^268^
- International organization: four studies^28^^,^^41^^,^^270^^,^^274^
- Patient advocacy group: two studies^25^^,^^266^
- For-profit organization: two studies^30^^,^^174^
- Collaboration across different type of organizations: 22 studies^2^^,^^19^^,^^29^^,^^53^^,^^65^^,^^66^^,^^68^^,^^121^^,^^124^^,^^125^^,^^145^^,^^150^^,^^152^^,^^168^^,^^191^^,^^227^^,^^235^^,^^236^^,^^248^^,^^251^^,^^256^^,^^269^^,^^272^
- Not specified or not applicable: 63 studies^12^^–^^14^^,^^21^^,^^22^^,^^26^^,^^31^^,^^36^^,^^37^^,^^42^^,^^43^^,^^48^^,^^56^^,^^59^^,^^60^^,^^67^^,^^70^^,^^73^^,^^75^^,^^76^^,^^87^^,^^89^^,^^93^^,^^111^^,^^127^^,^^136^^,^^146^^–^^148^^,^^154^^–^^161^^,^^163^^–^^165^^,^^176^^,^^179^^,^^190^^,^^193^^,^^199^^,^^202^^,^^208^^,^^213^^,^^217^^,^^218^^,^^226^^,^^229^^,^^230^^,^^232^^,^^249^^,^^250^^,^^253^^,^^257^^,^^261^^,^^262^^,^^264^^,^^267^^,^^273^^,^^277^

**Health system level of guideline**

- National: 168 studies^12^^,^^17^^–^^20^^,^^24^^,^^27^^,^^29^^,^^30^^,^^32^^–^^34^^,^^37^^,^^40^^,^^44^^–^^47^^,^^49^^,^^51^^,^^53^^,^^55^^–^^59^^,^^61^^–^^68^^,^^70^^,^^71^^,^^73^^,^^77^^–^^86^^,^^88^^,^^90^^,^^91^^,^^93^^–^^96^^,^^100^^,^^101^^,^^103^^,^^106^^–^^109^^,^^111^^–^^119^^,^^121^^–^^124^^,^^126^^,^^128^^,^^137^^,^^143^^,^^144^^,^^146^^–^^148^^,^^150^^,^^151^^,^^153^^–^^155^^,^^157^^–^^162^^,^^164^^,^^166^^,^^167^^,^^172^^,^^173^^,^^175^^–^^177^^,^^180^^–^^187^^,^^189^^–^^191^^,^^196^^,^^197^^,^^199^^,^^200^^,^^202^^–^^213^^,^^218^^–^^229^^,^^233^^,^^237^^–^^241^^,^^243^^–^^263^^,^^265^^,^^266^^,^^269^^,^^271^^–^^273^^,^^275^^,^^276^
- Multicountry: 50 studies^15^^,^^16^^,^^23^^,^^25^^,^^35^^,^^38^^,^^39^^,^^52^^,^^54^^,^^74^^,^^87^^,^^92^^,^^97^^–^^99^^,^^102^^,^^104^^,^^105^^,^^125^^,^^127^^,^^129^^–^^132^^,^^134^^–^^136^^,^^138^^–^^142^^,^^149^^,^^156^^,^^163^^,^^168^^,^^171^^,^^174^^,^^178^^,^^179^^,^^192^^,^^198^^,^^214^^–^^217^^,^^231^^,^^232^^,^^234^^,^^235^^,^^264^^,^^277^
- Global: 15 studies^2^^,^^28^^,^^41^^,^^50^^,^^69^^,^^110^^,^^145^^,^^188^^,^^194^^,^^195^^,^^201^^,^^236^^,^^242^^,^^270^^,^^274^
- Facility: eight studies^13^^,^^31^^,^^72^^,^^120^^,^^169^^,^^170^^,^^267^^,^^268^
- Regional: three studies^14^^,^^89^^,^^152^
- Not specified or not applicable: 14 studies^19^^,^^21^^,^^22^^,^^26^^,^^36^^,^^42^^,^^43^^,^^48^^,^^60^^,^^75^^,^^76^^,^^133^^,^^165^^,^^193^^,^^230^

**Income level of involved countries*^b^***

- High: 230 studies^2^^,^^12^^,^^13^^,^^15^^–^^48^^,^^50^^–^^72^^,^^74^^–^^77^^,^^79^^–^^113^^,^^115^^–^^119^^,^^121^^–^^125^^,^^127^^–^^152^^,^^155^^–^^159^^,^^161^^–^^164^^,^^166^^–^^192^^,^^195^^–^^207^^,^^209^^,^^210^^,^^212^^–^^215^^,^^217^^–^^223^^,^^225^^–^^241^^,^^243^^–^^247^^,^^249^^–^^259^^,^^262^^–^^269^^,^^276^^,^^277^
- Upper middle: 33 studies^2^^,^^25^^,^^28^^,^^36^^,^^41^^,^^49^^,^^50^^,^^73^^,^^78^^,^^92^^,^^110^^,^^114^^,^^120^^,^^126^^,^^153^^,^^154^^,^^160^^,^^165^^,^^171^^,^^192^^,^^198^^,^^201^^,^^211^^,^^224^^,^^230^^,^^234^^,^^248^^,^^260^^,^^270^^–^^273^^,^^275^
- Lower middle: 14 studies^2^^,^^14^^,^^28^^,^^41^^,^^50^^,^^110^^,^^171^^,^^192^^,^^198^^,^^201^^,^^208^^,^^230^^,^^261^^,^^270^
- Low: seven studies^2^^,^^28^^,^^41^^,^^50^^,^^110^^,^^201^^,^^230^
- Not specified: six studies^87^^,^^193^^,^^194^^,^^216^^,^^242^^,^^274^

^a^ Some studies were associated with more than one reviewed publication; the number of publications cited may therefore be greater than the number of identified studies in some cases. ^b^ For some studies, the target population and income level of involved countries fell within multiple categories.

We observed that the most common guideline topics were mental health or neurological conditions (50/258; 19.4%); general patient care and health promotion (35; 13.6%); musculoskeletal, connective tissue or immune conditions (25; 9.7%); circulatory or respiratory conditions (20; 7.8%); cancer (16; 6.2%); and genital or urinary system conditions (14; 5.4%). The target population of most guidelines were people affected by the guideline topics (242; 93.8%) or their caregivers (11; 4.3%).

We found that the most common guideline-issuing entities were professional associations, such as medical colleges or societies (73/258; 28.3%), nonprofit organizations (38; 14.7%) or government bodies (29; 11.2%). Most guidelines were developed at the national level (168; 65.1%) or multicountry level (50; 19.4%; typically multiple high-income countries), with few developed at the global level (15; 5.8%). Almost all guidelines involved populations of high-income countries in their development (230; 89.1%), with some also including upper-middle income (33; 12.8%) and lower-middle income (14; 5.4%) countries. Only seven guidelines involved people from low-income countries in their development (2.7%).

### Community engagement: characteristics and methods 

We report the characteristics and methods of community engagement described in the studies in Box 2. We observed that the communities that were engaged with were typically people directly affected by the topic (209/258, 81.0%), caregivers (98; 38.0%), patient advocates (18; 7.0%) or the public (21; 8.1%). We noted that the number of community members engaged in the guideline development varied widely from 2–10 people (39; 15.1%), 11–20 people (41; 15.9%), 21–50 people (41; 15.9%), 51–100 people (13; 5.0%) to more than 100 people (31; 12.0%).

Box 2 **Characteristics and methods of community engagement in studies^a^ identified in a methodological review of community engagement in guideline development**

**Type of community engaged (n = 258 studies)*^b^***

- People affected by the guidelines: 209 studies^12^^,^^13^^,^^15^^–^^24^^,^^26^^–^^30^^,^^32^^–^^38^^,^^40^^–^^51^^,^^53^^–^^68^^,^^70^^–^^72^^,^^74^^–^^82^^,^^84^^–^^90^^,^^93^^–^^96^^,^^98^^–^^107^^,^^110^^–^^114^^,^^116^^,^^119^^,^^121^^–^^129^^,^^131^^–^^134^^,^^136^^–^^139^^,^^141^^–^^159^^,^^162^^,^^164^^–^^169^^,^^171^^,^^173^^–^^176^^,^^178^^–^^180^^,^^183^^–^^192^^,^^194^^,^^196^^–^^199^^,^^202^^–^^205^^,^^207^^–^^212^^,^^214^^,^^216^^–^^231^^,^^233^^–^^243^^,^^249^^–^^253^^,^^258^^–^^260^^,^^263^^–^^267^^,^^269^^–^^273^^,^^275^^–^^277^
- Caregivers: 98 studies^13^^,^^14^^,^^17^^,^^20^^,^^25^^,^^30^^,^^32^^,^^34^^,^^37^^,^^40^^–^^45^^,^^47^^,^^48^^,^^55^^,^^60^^,^^62^^–^^66^^,^^68^^,^^69^^,^^73^^,^^75^^,^^76^^,^^79^^,^^87^^–^^89^^,^^91^^–^^93^^,^^98^^–^^100^^,^^103^^–^^106^^,^^113^^,^^115^^,^^119^^,^^121^^,^^125^^,^^126^^,^^129^^,^^131^^,^^132^^,^^134^^,^^138^^,^^142^^–^^144^^,^^146^^,^^148^^,^^151^^,^^157^^,^^158^^,^^160^^,^^161^^,^^163^^,^^164^^,^^170^^,^^181^^,^^182^^,^^185^^,^^186^^,^^195^^,^^197^^,^^198^^,^^203^^–^^205^^,^^208^^,^^212^^,^^217^^,^^224^^–^^226^^,^^230^^,^^233^^,^^236^^,^^240^^–^^246^^,^^251^^,^^257^^,^^258^^,^^261^^,^^262^^,^^264^^,^^266^^,^^268^^,^^276^^,^^277^
- Patient advocates: 34 studies^20^^,^^31^^,^^36^^–^^39^^,^^46^^,^^54^^,^^56^^,^^62^^,^^65^^,^^66^^,^^68^^,^^71^^,^^72^^,^^79^^,^^110^^,^^118^^,^^126^^,^^168^^,^^171^^,^^188^^,^^197^^,^^201^^,^^206^^,^^213^^,^^217^^,^^220^^,^^223^^,^^234^^–^^237^^,^^248^^,^^254^^,^^255^
- Public or general population: 21 studies^19^^,^^24^^,^^29^^,^^42^^,^^50^^,^^57^^,^^63^^,^^64^^,^^83^^,^^114^^,^^117^^,^^120^^,^^187^^,^^192^^,^^200^^,^^227^^,^^235^^,^^247^^,^^253^^,^^256^^,^^273^

**Number of members of community engaged (n = 258)**

- 2–10: 39 studies^16^^,^^18^^,^^23^^,^^27^^,^^31^^,^^45^^,^^49^^,^^56^^,^^81^^,^^82^^,^^88^^,^^101^^,^^107^^,^^111^^,^^118^^,^^127^^,^^128^^,^^151^^,^^154^^,^^161^^,^^169^^,^^171^^,^^175^^,^^185^^,^^186^^,^^195^^,^^197^^,^^200^^,^^201^^,^^204^^,^^213^^,^^214^^,^^221^^–^^223^^,^^237^^,^^248^^,^^259^^,^^266^
- 11–20: 41 studies^17^^,^^21^^,^^26^^,^^43^^,^^44^^,^^48^^,^^64^^,^^67^^,^^70^^,^^73^^,^^80^^,^^85^^,^^86^^,^^94^^–^^96^^,^^103^^,^^106^^,^^110^^,^^114^^,^^116^^,^^119^^,^^129^^,^^147^^,^^150^^,^^155^^,^^157^^,^^162^^,^^165^^,^^166^^,^^170^^,^^176^^,^^183^^,^^199^^,^^205^^,^^210^^,^^211^^,^^220^^,^^226^^,^^241^^,^^249^^,^^261^^,^^265^^,^^269^
- 21–50: 41 studies^12^^,^^14^^,^^24^^,^^25^^,^^28^^,^^30^^,^^40^^,^^42^^,^^55^^,^^58^^,^^60^^,^^62^^,^^75^^,^^76^^,^^89^^,^^91^^,^^104^^,^^121^^–^^123^^,^^141^^,^^142^^,^^146^^,^^148^^,^^156^^,^^159^^,^^167^^,^^178^^,^^181^^,^^196^^,^^198^^,^^212^^,^^230^^,^^239^^,^^240^^,^^250^^,^^253^^–^^257^^,^^263^^,^^264^^,^^277^
- 51–100: 13 studies^32^^,^^46^^,^^57^^,^^61^^,^^84^^,^^93^^,^^134^^,^^164^^,^^180^^,^^229^^,^^262^^,^^270^^,^^272^
- > 100: 31 studies^15^^,^^20^^,^^34^^,^^41^^,^^51^^,^^59^^,^^65^^,^^66^^,^^68^^,^^83^^,^^87^^,^^105^^,^^117^^,^^120^^,^^124^^,^^125^^,^^131^^,^^132^^,^^138^^,^^143^^,^^144^^,^^158^^,^^160^^,^^168^^,^^173^^,^^190^^,^^218^^,^^224^^,^^235^^,^^236^^,^^242^^,^^260^^,^^273^
- Not specified or not applicable: 93 studies^2^^,^^13^^,^^19^^,^^22^^,^^29^^,^^33^^,^^35^^–^^39^^,^^47^^,^^50^^,^^52^^–^^54^^,^^63^^,^^69^^,^^71^^,^^72^^,^^74^^,^^77^^–^^79^^,^^90^^,^^92^^,^^97^^,^^98^^,^^100^^,^^102^^,^^108^^,^^109^^,^^112^^,^^113^^,^^115^^,^^126^^,^^130^^,^^133^^,^^135^^–^^137^^,^^139^^,^^140^^,^^145^^,^^149^^,^^152^^,^^153^^,^^163^^,^^172^^,^^174^^,^^177^^,^^179^^,^^182^^,^^184^^,^^187^^–^^189^^,^^191^^–^^194^^,^^202^^,^^203^^,^^206^^–^^209^^,^^215^^–^^217^^,^^219^^,^^225^^,^^227^^,^^228^^,^^231^^–^^234^^,^^238^^,^^243^^–^^247^^,^^251^^,^^252^^,^^258^^,^^267^^,^^268^^,^^271^^,^^274^^–^^276^

**Method of engagement (n = 223)*^b^***

- Survey: 108 studies^15^^,^^18^^,^^20^^,^^24^^–^^26^^,^^28^^,^^30^^–^^32^^,^^37^^,^^38^^,^^40^^,^^41^^,^^44^^,^^46^^,^^49^^,^^51^^,^^56^^,^^59^^,^^61^^,^^62^^,^^64^^,^^65^^,^^67^^,^^78^^,^^79^^,^^81^^,^^83^^,^^84^^,^^87^^,^^88^^,^^91^^,^^100^^,^^102^^,^^104^^,^^105^^,^^110^^,^^112^^,^^114^^,^^117^^,^^120^^,^^121^^,^^124^^,^^125^^,^^129^^,^^131^^,^^132^^,^^134^^,^^138^^,^^139^^,^^141^^–^^146^^,^^153^^,^^154^^,^^157^^–^^159^^,^^161^^,^^164^^,^^168^^,^^170^^,^^173^^–^^176^^,^^181^^,^^182^^,^^185^^,^^186^^,^^188^^,^^190^^,^^192^^,^^198^^,^^199^^,^^201^^,^^212^^–^^214^^,^^218^^,^^224^^,^^227^^,^^229^^,^^233^^,^^235^^,^^236^^,^^238^^,^^239^^,^^242^^–^^247^^,^^254^^,^^255^^,^^260^^,^^262^^,^^263^^,^^267^^,^^270^^–^^273^^,^^275^^,^^277^
- Community representatives in guideline panel: 88 studies^15^^–^^18^^,^^20^^,^^23^^,^^24^^,^^27^^,^^29^^,^^37^^,^^38^^,^^41^^,^^44^^,^^46^^,^^48^^,^^50^^,^^56^^,^^57^^,^^62^^–^^66^^,^^68^^,^^70^^,^^71^^,^^74^^,^^80^^,^^82^^,^^85^^,^^86^^,^^90^^,^^96^^,^^101^^,^^107^^,^^112^^,^^114^^,^^122^^,^^123^^,^^126^^–^^128^^,^^137^^,^^139^^,^^141^^,^^146^^,^^154^^,^^157^^,^^159^^,^^161^^,^^162^^,^^168^^,^^171^^,^^181^^–^^183^^,^^186^^–^^188^^,^^191^^,^^192^^,^^194^^,^^198^^,^^201^^,^^203^^–^^205^^,^^208^^,^^210^^,^^211^^,^^218^^,^^220^^,^^223^^,^^228^^,^^231^^,^^235^^–^^237^^,^^243^^,^^244^^,^^248^^,^^249^^,^^253^^,^^259^^,^^263^^,^^266^^,^^268^^–^^270^^,^^273^^,^^276^
- Community workshops or focus groups: 79 studies^12^^,^^13^^,^^20^^,^^28^^–^^30^^,^^34^^,^^41^^,^^45^^,^^47^^,^^48^^,^^51^^,^^54^^,^^62^^,^^63^^,^^66^^,^^68^^,^^70^^,^^71^^,^^73^^,^^74^^,^^77^^,^^84^^,^^90^^,^^92^^,^^96^^,^^103^^,^^114^^–^^116^^,^^118^^,^^119^^,^^127^^,^^138^^,^^141^^,^^152^^,^^156^^,^^159^^,^^166^^,^^168^^,^^171^^,^^178^^,^^179^^,^^181^^,^^182^^,^^184^^,^^185^^,^^189^^,^^192^^,^^195^^–^^197^^,^^204^^,^^207^^,^^209^^,^^211^^,^^219^^,^^221^^–^^223^^,^^226^^,^^228^^,^^231^^,^^233^^,^^235^^,^^239^^–^^241^^,^^243^^,^^244^^,^^247^^,^^248^^,^^250^^,^^253^^,^^256^^,^^264^^–^^266^^,^^270^^,^^276^^,^^277^
- One-to-one meetings or interviews: 50 studies^12^^,^^13^^,^^34^^,^^45^^,^^55^^,^^60^^,^^65^^,^^66^^,^^68^^,^^70^^,^^75^^,^^76^^,^^89^^,^^93^^,^^101^^,^^115^^,^^116^^,^^118^^,^^120^^,^^124^^,^^136^^,^^146^^–^^148^^,^^150^^,^^153^^–^^155^^,^^160^^,^^165^^,^^169^^,^^178^^–^^182^^,^^185^^,^^186^^,^^197^^,^^202^^,^^208^^,^^229^^,^^243^^,^^250^^–^^253^^,^^257^^,^^261^^,^^264^^,^^267^^,^^268^^,^^273^
- Separate advisory group (no voting power): 35 studies^16^^,^^23^^,^^27^^,^^34^^,^^49^^,^^53^^,^^69^^,^^82^^,^^85^^–^^87^^,^^92^^,^^94^^–^^96^^,^^107^^,^^121^^–^^123^^,^^137^^,^^142^^,^^151^^,^^162^^,^^175^^,^^183^^,^^191^^,^^200^^,^^205^^,^^210^^,^^220^^,^^226^^,^^227^^,^^231^^,^^234^^,^^237^^,^^240^^,^^253^^–^^255^
- Lived-experience panel (voting power): 21 studies^17^^,^^32^^,^^40^^,^^56^^,^^62^^,^^79^^,^^81^^,^^88^^,^^121^^,^^129^^,^^134^^,^^143^^,^^144^^,^^164^^,^^167^^,^^171^^,^^176^^,^^197^^,^^214^^,^^238^^,^^258^
- Written feedback: 21 studies^13^^,^^29^^,^^34^^,^^38^^,^^53^^,^^54^^,^^64^^,^^118^^,^^126^^,^^157^^,^^171^^,^^197^^,^^225^^,^^231^^,^^236^^,^^240^^,^^245^^–^^247^^,^^253^^,^^273^
- Public comment and/or consultation: 21 studies^20^^,^^44^^,^^50^^,^^57^^,^^79^^,^^80^^,^^112^^,^^126^^,^^139^^,^^168^^,^^186^^–^^188^^,^^190^^,^^204^^,^^227^^,^^235^^,^^236^^,^^238^^,^^267^^,^^268^
- Training or support: 19 studies^13^^,^^14^^,^^20^^,^^24^^,^^70^^,^^85^^,^^86^^,^^94^^–^^96^^,^^114^^,^^128^^,^^167^^,^^180^^,^^194^^,^^231^^,^^236^^,^^239^^,^^260^^,^^271^^,^^275^
- Participatory workshops: 11 studies^14^^,^^20^^,^^72^^,^^111^^,^^120^^,^^147^^,^^168^^,^^179^^,^^180^^,^^235^^,^^265^
- Consulting with patient organizations: nine studies^70^^,^^74^^,^^114^^,^^197^^,^^226^^,^^235^^,^^236^^,^^253^^,^^273^
- Interactive tools: five studies^13^^,^^66^^,^^68^^,^^117^^,^^236^^,^^267^
- Online deliberation sessions: four studies^29^^,^^53^^,^^56^^,^^61^
- Nominal group technique: two studies^24^^,^^249^
- Listening event with public: two studies^57^^,^^225^
- Patient-run conference: one study^234^
- Writing group with community: one study^203^

**Community engagement and guideline development stage (n = 223 studies)*^b^***

- Define guideline scope: 83 studies ^13^^,^^15^^–^^18^^,^^20^^,^^24^^,^^30^^,^^37^^,^^41^^,^^44^^,^^46^^,^^55^^,^^56^^,^^59^^,^^60^^,^^63^^,^^65^^,^^66^^,^^68^^,^^70^^,^^72^^,^^74^^,^^79^^–^^81^^,^^84^^,^^90^^,^^92^^,^^96^^,^^100^^,^^103^^,^^104^^,^^110^^,^^114^^–^^116^^,^^119^^,^^120^^,^^125^^,^^131^^,^^132^^,^^137^^–^^139^^,^^147^^,^^154^^,^^157^^,^^166^^,^^168^^,^^173^^,^^175^^,^^179^^,^^180^^,^^186^^,^^188^^,^^191^^,^^192^^,^^204^^,^^205^^,^^209^^,^^220^^,^^223^^,^^227^^,^^229^^,^^231^^,^^233^^,^^235^^–^^237^^,^^240^^–^^242^^,^^248^^,^^254^^–^^256^^,^^259^^,^^262^^–^^267^^,^^269^^,^^270^^,^^277^
- Define community priorities, values, experiences or preferences: 179 studies^12^ ^,^^14^^–^^18^^,^^20^^,^^23^^,^^25^^,^^27^^–^^29^^,^^34^^,^^37^^,^^41^^,^^44^^–^^46^^,^^48^^–^^51^^,^^53^^,^^55^^,^^57^^,^^59^^,^^61^^,^^62^^,^^65^^–^^78^^,^^80^^–^^89^^,^^91^^–^^96^^,^^100^^–^^105^^,^^107^^,^^110^^–^^112^^,^^114^^–^^117^^,^^119^^–^^124^^,^^126^^–^^128^^,^^131^^,^^132^^,^^136^^,^^137^^,^^139^^,^^145^^–^^148^^,^^150^^–^^158^^,^^160^^–^^162^^,^^165^^–^^171^^,^^174^^,^^175^^,^^178^^–^^180^^,^^183^^–^^192^^,^^194^^–^^198^^,^^200^^–^^205^^,^^207^^,^^208^^,^^210^^,^^211^^,^^218^^–^^224^^,^^226^^–^^229^^,^^231^^,^^233^^,^^235^^,^^236^^,^^238^^–^^246^^,^^250^^,^^251^^,^^253^^–^^258^^,^^260^^–^^272^^,^^275^^–^^277^
- Review evidence and/or formulate recommendations: 114 studies ^12^^,^^15^^–^^18^^,^^20^^,^^23^^,^^24^^,^^26^^,^^27^^,^^29^^,^^31^^,^^32^^,^^37^^,^^38^^,^^40^^,^^41^^,^^44^^,^^46^^,^^48^^,^^50^^,^^56^^,^^62^^–^^66^^,^^68^^,^^70^^,^^71^^,^^74^^,^^80^^–^^82^^,^^85^^,^^86^^,^^90^^,^^92^^,^^94^^–^^96^^,^^101^^,^^107^^,^^110^^–^^112^^,^^114^^,^^120^^–^^123^^,^^126^^–^^129^^,^^134^^,^^137^^,^^139^^,^^141^^,^^146^^,^^147^^,^^154^^,^^157^^,^^159^^,^^161^^,^^162^^,^^164^^,^^167^^,^^168^^,^^171^^,^^176^^,^^180^^–^^183^^,^^185^^–^^187^^,^^191^^,^^192^^,^^194^^,^^198^^,^^201^^,^^203^^–^^205^^,^^208^^,^^210^^,^^213^^,^^214^^,^^218^^,^^220^^,^^221^^,^^223^^,^^227^^–^^229^^,^^231^^,^^235^^–^^237^^,^^240^^,^^242^^–^^244^^,^^248^^,^^249^^,^^253^^,^^258^^,^^263^^,^^265^^–^^270^^,^^276^^,^^277^
- Review drafted recommendations: 137 studies ^13^^–^^18^^,^^20^^,^^23^^,^^24^^,^^27^^,^^29^^,^^34^^,^^37^^,^^38^^,^^40^^,^^41^^,^^44^^,^^48^^,^^50^^,^^53^^,^^54^^,^^56^^,^^57^^,^^59^^,^^62^^–^^68^^,^^70^^–^^72^^,^^74^^,^^77^^,^^79^^–^^82^^,^^85^^,^^86^^,^^90^^,^^92^^,^^96^^,^^101^^,^^103^^,^^107^^,^^110^^–^^112^^,^^114^^,^^116^^–^^118^^,^^120^^–^^123^^,^^126^^–^^129^^,^^131^^,^^132^^,^^134^^,^^137^^,^^139^^,^^141^^–^^144^^,^^146^^,^^151^^,^^154^^,^^157^^,^^159^^,^^161^^,^^162^^,^^167^^,^^168^^,^^171^^,^^179^^,^^181^^–^^183^^,^^185^^–^^188^^,^^190^^–^^192^^,^^194^^,^^197^^–^^205^^,^^208^^,^^210^^,^^214^^,^^218^^,^^220^^,^^223^^,^^225^^,^^227^^,^^231^^,^^234^^–^^237^^,^^239^^,^^240^^,^^242^^–^^249^^,^^253^^–^^256^^,^^258^^,^^259^^,^^263^^,^^266^^–^^270^^,^^276^
- Create community version of guidelines: 19 studies^14^^,^^29^^,^^32^^,^^34^^,^^38^^,^^40^^,^^70^^,^^74^^,^^87^^,^^101^^,^^103^^,^^114^^,^^126^^,^^139^^,^^196^^,^^231^^,^^235^^,^^236^^,^^273^
- Implement and/or disseminate guidelines: 15 studies^29^^,^^48^^,^^63^^,^^70^^,^^74^^,^^92^^,^^114^^,^^117^^,^^126^^,^^168^^,^^181^^,^^192^^,^^234^^–^^236^

**Method of evaluation of community engagement (n = 223 studies)*^b^***

- Interview: eight studies^66^^,^^68^^,^^114^^,^^117^^,^^131^^,^^132^^,^^196^^,^^197^^,^^253^^,^^259^
- Survey: eight studies^12^^,^^24^^,^^66^^,^^68^^,^^114^^,^^117^^,^^159^^,^^196^^,^^240^
- Document review: four studies^196^^,^^197^^,^^241^^,^^253^
- Direct observation: two studies^196^^,^^253^
- None: 210 studies^13^^–^^18^^,^^20^^,^^23^^,^^25^^–^^32^^,^^34^^,^^37^^,^^38^^,^^40^^,^^41^^,^^44^^–^^51^^,^^53^^–^^57^^,^^59^^–^^65^^,^^67^^,^^69^^–^^96^^,^^100^^–^^105^^,^^107^^,^^110^^–^^112^^,^^115^^,^^116^^,^^118^^–^^129^^,^^134^^,^^136^^–^^139^^,^^141^^–^^148^^,^^150^^–^^158^^,^^160^^–^^162^^,^^164^^–^^171^^,^^173^^–^^176^^,^^178^^–^^192^^,^^194^^,^^195^^,^^198^^–^^205^^,^^207^^–^^214^^,^^218^^–^^229^^,^^231^^,^^233^^–^^239^^,^^242^^–^^251^^,^^254^^–^^258^^,^^260^^–^^277^

^a^ Some studies were associated with more than one reviewed publication; the number of publications cited may therefore be greater than the number of identified studies in some cases. ^b^ For some studies, the community engagement characteristics fell within multiple categories.

**Fig. 2:**
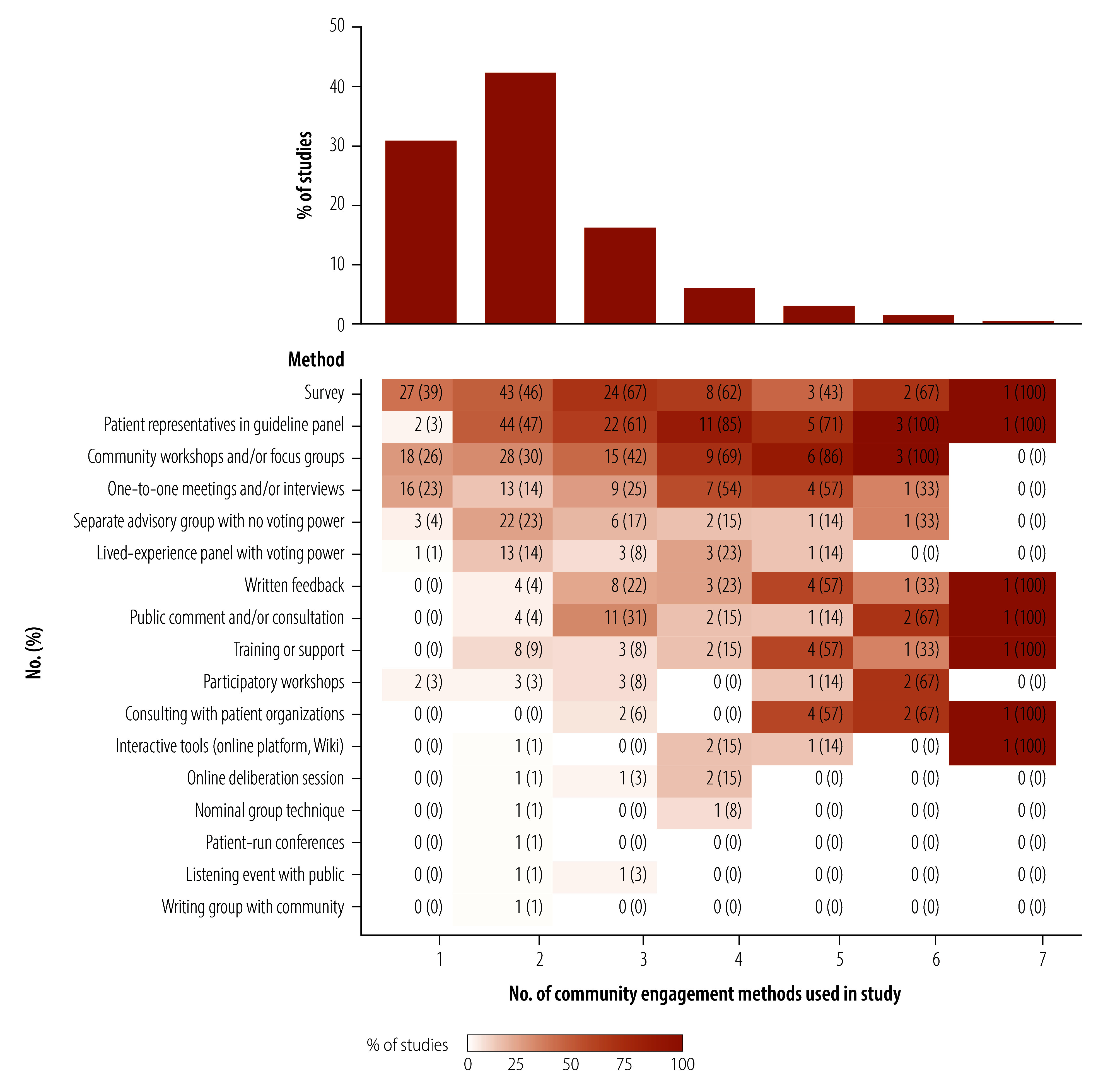
Percentage of studies reporting the use of different methods of community engagement in guideline development

Notes: the denominator is 223 publications. In the lower graph, the total percentages increase with the number of methods used by study, for example, the column presenting three methods will show a total of 300%. To determine the number of studies using a given number of methods, the total count of methods used needs to be divided by that number.

A total of 223 studies described methods of community engagement, and we noted that 17 different methods of community engagement were used. Most guidelines (154/223; 69.1%) used more than one method of engagement. The most common methods were: surveys (108; 48.4%), community representatives on the guideline panel (88; 39.5%), community workshops (79; 35.4%) and one-to-one meetings or interviews (50; 22.4%). Some convened community advisory groups with no voting power (35; 15.7%) or lived-experience panels with voting power (21; 9.4%). Written feedback, public comment or consultation, training or support, participatory workshops, consulting with patient and/or community organizations, and interactive tools were less common (less than one tenth), typically in combination with two or more other methods (Fig. 2). Online deliberation sessions, nominal group techniques, patient-run conferences, listening events with the public and community writing groups were described in the publications of four or fewer unique studies.

We depict the distribution of community engagement methods based on the total number of methods used in a guideline in Fig. 2. We observed that the engagement methods used were more likely to engage people with lived experience or caregivers as opposed to the general public. When the general public was engaged, we noted that it was typically in guideline development involving three or more methods.

### Community engagement: guideline development stages

A total of 223 unique studies described community engagement or evaluation methods across the six stages of guideline development, namely: defining scope (83/223; 37.2%); defining community priorities (179; 80.3%); reviewing evidence and/or formulating recommendations (114; 51.1%); reviewing drafted recommendations (137; 61.4%); creating a community version (19; 8.5%); or implementing or disseminating the guideline (15; 6.7%; Box 2). 

Approximately one half of the studies engaged communities across three or more stages of the guideline development, typically describing community priorities, values, experiences or preferences (online repository).^11^

### Evaluation of community engagement 

Thirteen studies described the evaluation of community engagement in guideline development, using interviews (8/223; 3.6%), surveys (8; 3.6%), document review (4; 1.8%) or direct observations of the guideline process (2; 0.9%; Box 2). One study described an evaluation of the reliability and validity of the patient engagement evaluation tool, which assessed trust, respect, fairness, competency, legitimacy and accountability.^172^ The authors proposed a valid and reliable six-item Likert scale using items including “to what extent do you believe your ideas were heard during the engagement process?”^172^

### Learning from best practices 

Some publications reflected on best practices or innovations in guideline development, or explained what worked and did not work, and why.^21^^,^^33^^,^^35^^,^^39^^,^^43^^,^^52^^,^^58^^,^^98^^,^^106^^,^^109^^,^^130^^,^^132^^,^^133^^,^^140^^,^^177^^,^^230^^–^^232^^,^^274^ Foundational to meaningful community engagement in guideline development was strategic organizational commitment via formal strategies, resources and established links.^35^^,^^97^^,^^109^^,^^135^^,^^230^ We learnt that recruitment strategies for community members should be planned and targeted and, when conducted properly, could help to achieve diversity.^39^^,^^43^^,^^97^^,^^231^^,^^232^ Community members could be involved in nominating and selecting health expert guideline development group members.^52^^,^^98^^,^^133^ Given group power dynamics, several recommended at least two (ideally more) community representatives on guideline development groups to encourage participation^21^^,^^97^^,^^106^^,^^109^^,^^215^^,^^232^ and to make room for clients and family to make a worthy contribution.^21^^,^^97^^,^^106^^,^^109^^,^^232^ Where community members were also members of the guideline development group, strong chairing had the potential to encourage active and equitable participation from all.^21^^,^^215^^,^^231^^,^^232^

Several studies described the important role of training and support for community representatives throughout the guideline development process, including orientation, guideline method training, informal support (e.g. pre-meeting briefings) and accessibility accommodations (e.g. live-captioning online meetings, and careful consideration of the language of guideline development to avoid excluding anyone).^21^^,^^33^^,^^39^^,^^43^^,^^52^^,^^58^^,^^97^^,^^140^^,^^215^^,^^231^^,^^232^^,^^254^ Such training and partnership had the capacity to shape a shared vision for the future^109^ and brought clarity to community member roles and objectives, which helped to manage expectations.^52^^,^^97^^,^^132^^,^^215^^,^^231^^,^^232^ Training health expert or professional guideline development group members on effective collaboration with community representatives was observed to be critical.^130^ Financial compensation for attendance and/or sitting fees, as well as reimbursement of expenses, helped to improve equity and reduce the burden of participation.^132^^,^^215^^,^^231^^,^^232^ Certificates of appreciation or formal acknowledgement of participation could be a nonfinancial motivator for community engagement.^231^^,^^232^

One substantive mechanism for community engagement observed was to convene a separate lived-experience panel with voting power, comprising people with lived experience of the heath condition or carers. This panel met separately from the guideline development group, and were involved at all stages of the guideline development. Typically, their feedback and decisions fed into guideline development group deliberations, with several members of the lived-experience panel also sitting on the guideline development group. Given power dynamics favouring clinician dominance in guideline development,^135^^,^^215^ such substantive approaches to community engagement may help to shift the norm. Community members often feel overlooked – as one participant in a study on child participation in guideline development expressed, “as a representative of the patient perspective, you remain a player in someone else’s game”.^109^ Collectively, these more substantive approaches to community engagement necessitate flexibility, sufficient time and funding, and the creation of space and time for meaningful engagement.^109^^,^^135^^,^^215^

Innovative methods for community engagement in dissemination include public meetings, visual story-telling, science promotion events, project websites, interactive workshops and advocacy platforms.^97^^,^^135^^,^^230^ Beyond dissemination, community engagement approaches to guideline implementation include co-design workshops to develop implementation strategies, community-led information sessions and web-based resources, such as virtual patient feedback platforms, podcasts and videos.^97^^,^^230^ Box 2 and the online repository^11^ highlight how these dissemination and implementation approaches appear to be underutilized, despite offering a rich opportunity to improve guideline adoption.

## Discussion

The findings of our review have significant implications for community engagement in guideline development. Current practices typically involve limited engagement, but there exists significant potential to enhance the relevance, acceptability and impact of guidelines through substantive community engagement. Although many guideline development groups (including those of WHO) currently include only one to two community representatives, our review highlights strategies for a more comprehensive and innovative engagement with the community. 

We found very few examples of guideline development with international reach and community engagement, raising questions about what effective global community engagement could look like. Achieving meaningful community engagement at a global level requires that complex issues of geographical representation, language inclusion and logistical constraints be addressed, and presents unique challenges for organizations that develop global norms and standards. For organizations with a global mandate, such as WHO, innovative approaches, such as online communities of practice and platforms, will be needed to overcome these challenges and ensure diverse community voices are represented throughout guideline development. Guideline-issuing organizations will therefore need to reconcile inclusivity with feasibility of community engagement, including resource use; such considerations were rarely reported in the reviewed publications. Achieving such a balance will be particularly challenging, particularly in the absence of evidence-based approaches to balance inclusivity and feasibility. Documenting experiences and results as case studies is advisable for dissemination and continuous learning. Sustainability must also be considered, and funders must commit to community engagement.

Expanding beyond token representation of the community to the substantive engagement methods identified in our review, such as lived-experience panels or community advisory groups, could enhance the relevance and impact of guidelines. However, this requires careful consideration of funding and logistical constraints. Remunerating community members for their time and expertise in global guideline work is costly. Technological barriers, such as unreliable internet, may impede virtual engagement that could otherwise help to overcome geographical limitations. Despite these challenges, finding ways to meaningfully involve diverse community voices around the world in guideline development remains an important goal. Innovative approaches tailored to the context of the guideline-issuing organization are needed to achieve comprehensive community engagement, while balancing practical constraints.

Although we identified a tool to evaluate community engagement,^172^ an internationally accepted approach to evaluating community engagement in guidelines is currently lacking. As commitments are made to better and more meaningful community engagement, tools to measure and evaluate dimensions of engagement and impact are needed. Moreover, approximately one third of our reviewed publications did not report the number of community members engaged (proxy for reach of engagement). Additional tools are needed to evaluate the reach and breadth of community engagement, particularly for global entities such as WHO.

Our methodological review to map existing practices of community engagement in the development or adaptation of health guidelines benefited from the use of robust methods to identify areas in which community engagement approaches could be developed or strengthened. 

Our approach has some limitations. Incomplete reporting in certain domains may result in underreporting, and some descriptions of community engagement across guideline development stages lacked detail. We focused on community engagement, but further research may be necessary to more accurately represent the inclusion of other stakeholder groups, such as payers, purchasers and product makers,^2^^,^^193^ in guideline development.

Our review highlights the importance and challenges of implementing community engagement in global health guideline development. This process may involve adapting existing engagement methods to a global context, adapting methods used in primary research, leveraging technology while being mindful of access limitations, and carefully balancing the costs and benefits of extensive engagement. Ultimately, striving for inclusive and participatory guideline development processes can lead to effective and equitable health recommendations worldwide.
